# The FDA-approved drug Auranofin has a dual inhibitory effect on SARS-CoV-2 entry and NF-κB signaling

**DOI:** 10.1016/j.isci.2022.105066

**Published:** 2022-09-03

**Authors:** Emmanuel Laplantine, Christine Chable-Bessia, Anne Oudin, Jitendryia Swain, Adèle Soria, Peggy Merida, Manon Gourdelier, Sarra Mestiri, Indira Besseghe, Erwan Bremaud, Aymeric Neyret, Sebastien Lyonnais, Cyril Favard, Philippe Benaroch, Mathieu Hubert, Olivier Schwartz, Maryse Guerin, Anne Danckaert, Elaine Del Nery, Delphine Muriaux, Robert Weil

**Affiliations:** 1 Sorbonne Universités, Institut National de la Santé et de la Recherche Médicale (INSERM, UMR1135), Centre National de la Recherche Scientifique (CNRS, ERL8255), Centre d’Immunologie et des Maladies Infectieuses CMI, Paris, France; 2 CEMIPAI, Montpellier University, UAR3725 CNRS, Montpellier, France; 3 Institute of Research in Infectiology of Montpellier (IRIM), University of Montpellier, UMR9004 CNRS, Montpellier, France; 4 Institut Curie, PSL Research University, Department of Translational Research-Biophenics High-Content Screening Laboratory, Cell and Tissue Imaging Facility (PICT-IBiSA), 75005 Paris, France; 5 Institut Curie, PSL University, Inserm U932, Immunity and Cancer, 75005 Paris, France; 6 Institut Pasteur, Virus and Immunity Unit, Department of Virology, Paris, France; 7 Centre National de la Recherche Scientifique (CNRS, UMR3569), Paris, France; 8 National Institute for Health and Medical Research (INSERM) UMRS 1166, Faculty of Medicine Pitié-Salpêtrière, 91 Bld de L'Hôpital, 75013 Paris, France; 9 Institut Pasteur, UTechS Photonic BioImaging (PBI) – C2RT, Paris, France

**Keywords:** Chemistry, Biochemistry, Medical biochemistry, Molecular biology

## Abstract

Patients with severe COVID-19 show an altered immune response that fails to control the viral spread and suffer from exacerbated inflammatory response, which eventually can lead to death. A major challenge is to develop an effective treatment for COVID-19. NF-κB is a major player in innate immunity and inflammatory process. By a high-throughput screening approach, we identified FDA-approved compounds that inhibit the NF-κB pathway and thus dampen inflammation*.* Among these, we show that Auranofin prevents post-translational modifications of NF-κB effectors and their recruitment into activating complexes in response to SARS-CoV-2 infection or cytokine stimulation. In addition, we demonstrate that Auranofin counteracts several steps of SARS-CoV-2 infection. First, it inhibits a raft-dependent endocytic pathway involved in SARS-CoV-2 entry into host cells; Second, Auranofin alters the ACE2 mobility at the plasma membrane. Overall, Auranofin should prevent SARS-CoV-2 infection and inflammatory damages, offering new opportunities as a repurposable drug candidate to treat COVID-19.

## Highlights

• Original high throughput screening of NF-κB inhibitory drugs
• Auranofin inhibits SARS-CoV-2 replication
• Auranofin increases the ACE2 mobility at the plasma membrane
• Auranofin inhibits ACE-2-dependent SARS-CoV-2 endocytosis

## Graphical abstract

**Figure:**
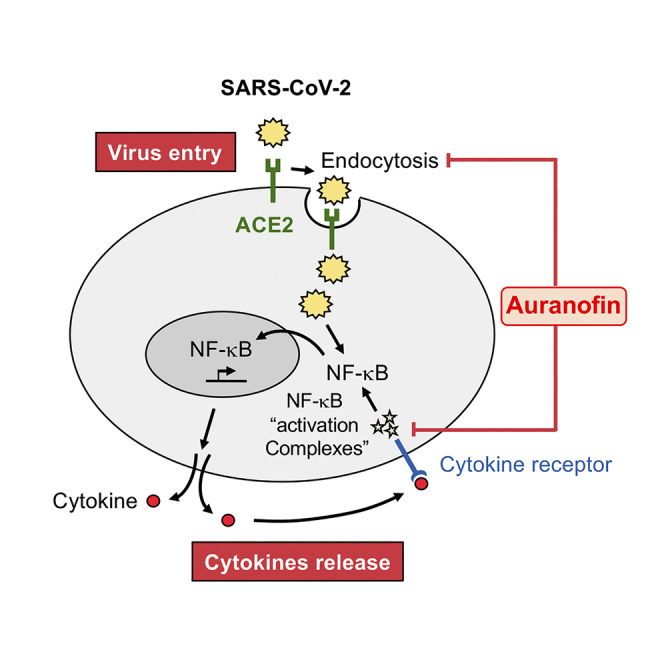

## Introduction

SARS-CoV-2 virus has infected almost 340 million people worldwide and killed almost 6 million to date. The majority of infected people experience only mild or moderate symptoms and recover without special treatment. However, about 20% of those infected have inflammatory lung disease with upper airway damage, fever, and dyspnea. Patients usually recover in 2 weeks for mild forms and 3–6 weeks for severe forms. In sum, 5% of patients are hospitalized in intensive care with respiratory distress. For these patients, inflammatory processes affect multiple organs besides the lungs, leading to heart, liver, and kidney failures. In addition, the strong release of cytokines by the immune system in response to viral infection and/or secondary infections induces a “cytokine storm” and sepsis, which are the major causes of death.

A key step in infection is the binding of the viral protein to its receptor, the angiotensin-converting enzyme 2 (ACE2), at the host cell surface (airway epithelial cells, vascular endothelial cells, and pulmonary macrophages). The virus entry is facilitated by the interaction of its Spike (S) with heparan sulfate (HS) and the cleavage of S protein at the S1-S2 junction by Furin and the cellular serine protease TMPRSS2 (Clausen et al., 2020; Coutard et al., 2020; Hoffmann et al., 2020). These enzymatic processes allow its binding to another host factor, the membrane protein neuropilin-1 (NRP1), and merging with cell membranes, respectively (Cantuti-Castelvetri et al., 2020; Daly et al., 2020). Coronavirus can enter host cells through the membrane fusion of the virus envelope with the plasma membrane when TMPRSS2 is expressed (Lukassen et al., 2020; Murgolo et al., 2021). Alternatively, the virus can use an endocytic pathway within the endosomal-lysosomal compartments involving processing by the lysosomal Cathepsin L (Ou et al., 2020). As for other coronaviruses, the cholesterol in lipid rafts and endosomal acidification is indispensable for SARS-CoV-2 replication (Lu et al., 2008). Faced with the COVID-19 pandemic, expectations for an efficient preventive and curative treatment are high. Several molecular pathways have been evaluated to find COVID-19 treatments based on strategies that have been already considered for other viruses. Since the onset of the epidemic, Remdesivir (Veklury, Gilead laboratory), a nucleotide analog targeting the viral RNA-dependent RNA polymerase (RdRp) and originally developed for the treatment of Ebola virus, rapidly obtained marketing authorization for the treatment of COVID-19 patients receiving oxygen therapy before the World Health Organization (WHO) advises against its use (Beigel et al., 2020). Given the emergency, many studies were carried out using already approved treatments. For instance, the UK clinical trial Recovery has shown the efficiency of Dexamethasone, a steroidal anti-inflammatory drug, on severe forms with elevated C-reactive protein levels of COVID-19 (Calzetta et al., 2021). More recently, Merck-MSD and Pfizer have developed specific SARS-CoV-2 antiviral treatments, named Lagevrio and Paxlovid, respectively. These drugs are expected to reduce hospital admissions although their real impact will only be assessed by large-scale clinical trials. However, the efficiency of these drugs against all variants of SARS-CoV-2 is not known. Besides these antiviral treatments, several laboratories offer specific monoclonal antibodies intended for the early treatment of mild to moderate forms of COVID-19: Xevudy (GSK and Vir Biotechnology); AZD7442 (Astrazeneca); bamlanivimab/etesevimab (Lilly); casirivimab/imdevimab (Regeneron). Issues related to these treatments reside in their very high cost and their administration mode. Other treatments specifically targeting SARS-CoV-2 are thus under current investigation and are required to prevent virus dissemination, inflammation, and long COVID-19 symptoms. NF-κB inhibitors represent avenues to prevent the exacerbated inflammation caused by the vigorous immune response with the high secretion of cytokines, a hallmark of severe COVID-19 forms. To find new inhibitors of NF-κB, we developed a high-throughput screening of the FDA-approved drug library Prestwick chemical based on the inhibition of cytokine-induced supramolecular complexes involved in NF-κB activation (Tarantino et al., 2014). This screen led to the identification of 60 candidates among which four major “hits” reached a high confidence score by statistical analyses. Auranofin, a gold salt with anti-inflammatory properties, was the strongest hit identified. Auranofin exhibits a metal ion (Gold) that interacts with enzymes containing sulfur- or nitrogen groups, notably with their thiol or imidazole groups. It has been proposed that Auranofin interacts with a sulfhydryl group of IKK that is critical for its enzymatic activity but our results suggest that Auranofin rather inhibits a more upstream enzyme (Jeon et al., 2000). Interestingly, we also found in our screen Thimerosal which is a mercury-containing compound that might similarly target enzyme(s) of the NF-κB pathway that contain free thiols. Auranofin was approved 33 years ago by the FDA for the treatment of rheumatoid arthritis and is currently in phases II and III for clinical trials in cancer therapy (Finkelstein et al., 1976). In addition, gold-based compounds have shown promising activity against several microbial infections including viruses (HIV), parasites, and bacteria (Diaz et al., 2019; Fuchs et al., 2016). Auranofin is administered orally at 6 mg daily (at a single dose or 3 mg twice a day) and displays few long-term side effects.

Interestingly, Auranofin was initially proposed as a putative treatment of COVID-19 (Marzo and Messori, 2020) and, thereafter, two studies showed that Auranofin inhibits SARS-CoV-2 replication and inflammatory cytokine expression (IL-6, TNF-α, and IL-1β) in Huh-7 cells and at lower concentrations in HEK-ACE2 and Vero E6 cells, whereas a recent study claimed that Auranofin has no effect on SARS-CoV-2 replication in Calu-3 cells (Biji et al., 2021; Cirri et al., 2021; Rothan et al., 2020). Moreover, oral administration of Auranofin in Syrian hamsters in therapeutic and prophylactic regimen was shown to reduce lung tissue damage, cellular infiltration, inflammation, and IL-6 production (Biji et al., 2021). Recently, hundreds of structurally diverse metal complexes have been investigated against the S/ACE2 interaction and the papain-like protease of SARS-CoV-2 (PLpro) (Gil-Moles et al., 2021). Whereas this study showed that Auranofin has no obvious effect on the S/ACE2 interaction, it displayed a strong inhibitory effect on PLpro, which also interferes with the interferon response by cleaving ISGylated proteins (Gil-Moles et al., 2020).

Here we demonstrated that Auranofin is a potent inhibitor of the NF-κB pathway in response to multiple stimuli including the proinflammatory cytokines IL-1 and TNFα and SARS-CoV-2 infection. Auranofin prevents the formation of activation complexes containing the NF-κB essential modulator (NEMO) in response to IL-1, TNF, and LPS. Data from the literature suggest that Auranofin may be a catalytic inhibitor of IKK kinases (Jeon et al., 2003). However, we found that Auranofin acts upstream in the NF-κB signaling cascade. A comparative analysis of the different compounds that were identified in our screen showed that only Auranofin protects from SARS-CoV-2 cellular entry in various cell lines and that it reduces the number of replication complexes *in vitro*. Mechanistically, we found that Auranofin inhibits the raft-dependent endocytic pathway and increases ACE2 mobility at the cell surface thus impairing SARS-CoV-2 cell entry. Altogether, we provide the mechanisms by which Auranofin inhibits SARS-CoV-2 entry and NF-κB activation, which could be beneficial in treating multi-organ systemic inflammation that can lead to death.

## Results

### Identification of Auranofin as an NF-κB supramolecular complex inhibitor

**Figure 1:**
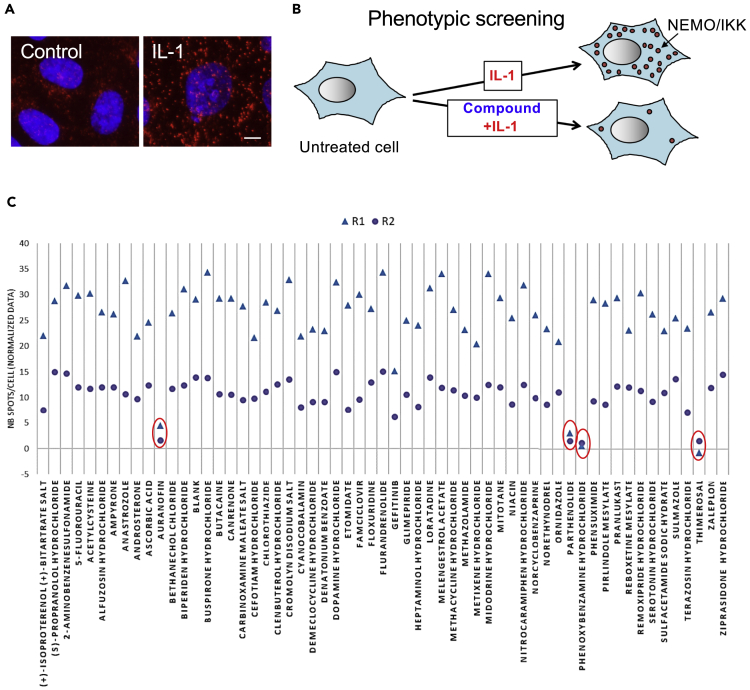
Chemical compound screening interfering with the formation of supramolecular complexes (A) Immunofluorescence detection of NEMO in untreated and IL-1-treated U2OS cells. Scale bar, 5 μm. (B) The screen aims at identifying chemical compounds that interfere with the formation of supramolecular complexes induced by IL-1, which are correlated with the activation of NF-κB. (C) Hit validation scores: graphical representation of the normalized number of dots per cell compared with negative controls to validate hits based on SSMD score for 60 compounds (in duplicate R1 and R2).

Our laboratory previously showed that the proinflammatory cytokines, interleukin 1 (IL-1) and TNF-α, induce the rapid and transient recruitment of NEMO and active forms of IKK kinases at the level of intracellular supramolecular structures (Tarantino et al., 2014). The formation of these structures correlates with NF-κB activation. Detection of NEMO-containing complexes allows a high content screening strategy aiming at identifying chemical compounds that interfere with their formation, proceeding, and required for NF-κB activation (Figure 1A). The chemical screen was carried out with a collection of 1,270 off-patent drugs that are fully approved by the FDA for specific therapeutic indications and are particularly suitable for finding new targets (an approach called “repositioning”). The screen led to the identification of four hit compounds with a high confidence score as calculated by statistical analyses and independently re-validated in a second screen (Figure 1B).

**Figure 2:**
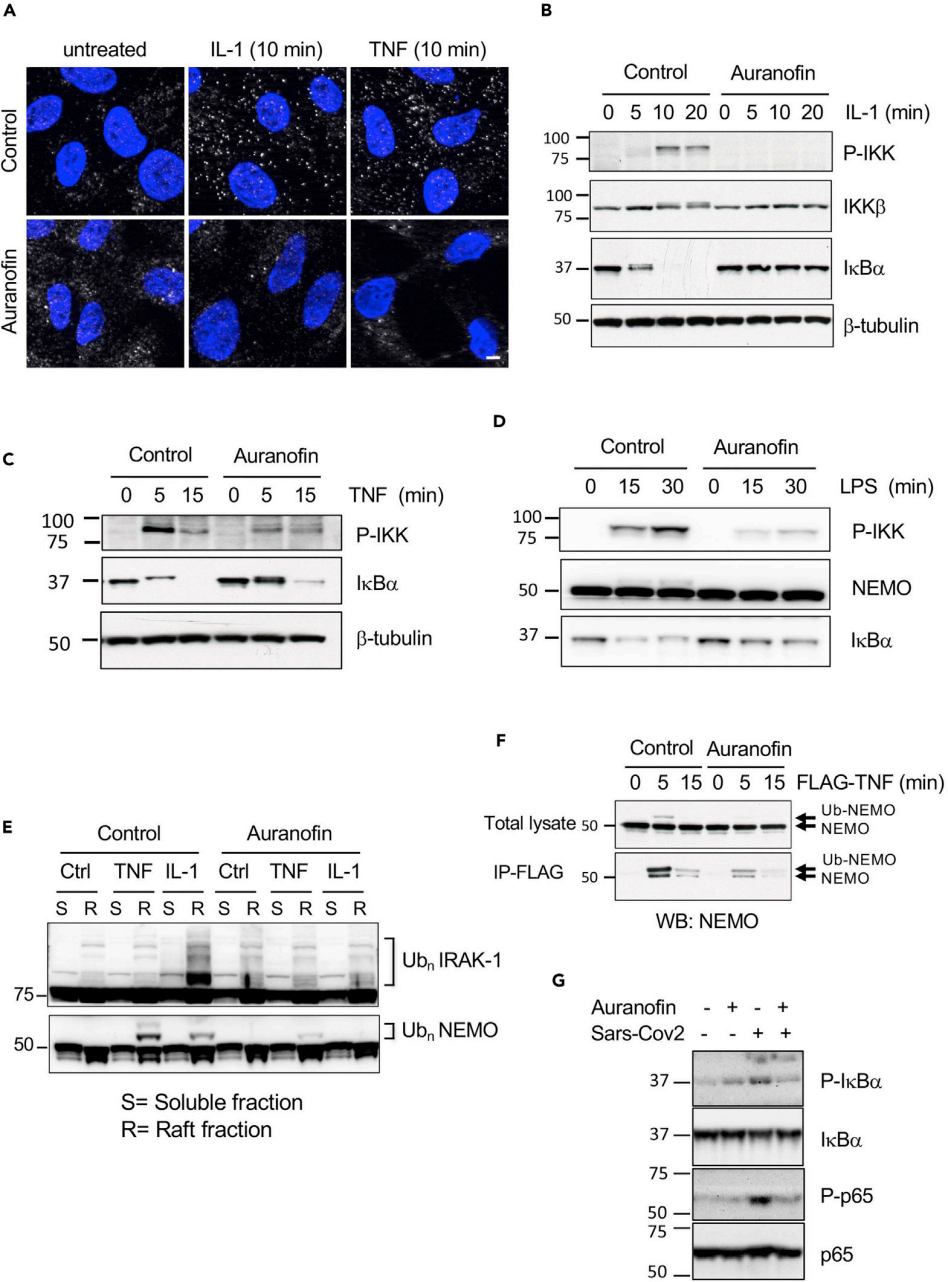
Auranofin inhibits NF-κB activation (A) Auranofin inhibits TNF-α and IL1-mediated accumulation of NEMO in punctuates structures. Immunofluorescence analysis of NEMO recruitment in supramolecular complexes in U2OS cells untreated or pretreated with Auranofin (5μM for 90 min) and stimulated with IL-1 or TNF-α for 10 min. Scale bar, 5 μm. (B, C, and D) Auranofin inhibits IKK phosphorylation and IκBα degradation. U2OS cells were treated with IL-1 (B), TNF-α (C), or LPS (D) either alone or in association with Auranofin (10 μM). Cell lysates were analyzed by Western blot using anti-phospho-IKK, anti-IκBα, or anti-β-tubulin antibodies (loading control). (E) Auranofin inhibits TNF-α and IL1-induced NEMO and IRAK1 ubiquitination. U2OS cells were pretreated with Auranofin as indicated and left unstimulated (Ctrl) or stimulated with TNF-α or IL-1. After lysis in 1% TX100-containing lysis buffer and removal of the nuclei, lysates were submitted to an ultracentrifugation in order to collect soluble (S) and insoluble raft fractions (R), followed by western blotting analyses with antibodies against IRAK1, NEMO. The ubiquitinated forms of these proteins are indicated on the right. (F) Auranofin prevents NEMO recruitment to the TNF-RSC in response to IL-1 and TNF-α. Kinetic analysis of TNF-RSC formation induced by FLAG-TNF stimulation. U2OS cells, pre-treated or not (control) with Auranofin were stimulated with FLAG-TNF for the indicated time and immunoprecipitated by anti-FLAG antibodies. The isolated protein complexes were immunoblotted to detect the presence of unmodified and ubiquitinated forms of NEMO as indicated. (G) Auranofin inhibits NF-κB activation in response to SARS-CoV-2 infection. CALU-3 cells were pre-incubated with 2.5-μM Auranofin for 90 min before infection with SARS-CoV-2 at MOI 0.05 for 48 h. Cell lysates were analyzed by Western blot using anti-phospho-p65, anti-phospho-IκBα, anti-p65, and anti-IκBα. See also Figures S1–S4.

We focused our attention on Auranofin as this compound is currently being revisited in many infectious diseases and cancers (Table 1). We first confirmed by immunofluorescence microscopy that Auranofin prevents the formation of NEMO-containing supramolecular complexes in response to IL-1, but also in response to TNF-α (Figure 2A). This effect of Auranofin was neither owing to an inhibitory effect of Auranofin on TNF-R and IL1-R expression (Figure S1) nor a direct effect of Auranofin on IKK activity, as previously suggested (Jeon et al., 2003), as these activation complexes were not inhibited by ML120B, a conventional NF-κB inhibitor (Figure S2A), despite their inhibitory effects on IL-1 and TNF-α- mediated p65 nuclear translocation (Figure S2B). We also observed that the interleukin-1 receptor-associated kinase 1 (IRAK1), a kinase that plays an important role in IL-1-dependent NF-κB activation and innate immunity against pathogens, was not detected in supramolecular complexes when Auranofin was added before IL-1 stimulation (Figure S3).

**Table 1:** Top 4 compounds inhibiting NF-κB activation

Compound name	Structure	Known targets	Therapeutic use
Auranofin		Thioredoxin reductase IKK	Arthritis Anti-cancer (under study)
Parthenolide		Redox enzymes IKK	Arthritis, headache
Phenoxybenzamine		α-adrenergic receptor	Hypertension Vasodilator
Thimerosal		ND	Vaccine preservative (antiseptic)

Top 4 drugs that strongly reduce the formation of NEMO-containing supramolecular complexes needed for NF-κB activation. The inhibitory effect of these four drugs on NF-κB was confirmed and validated after the initial screening by imaging approaches and biochemical assays.

To examine the expression of phosphorylated IKK and IκBα by western blot, U2OS and THP1 cells were pretreated with Auranofin and then stimulated with IL-1, TNFα, or LPS. In these conditions, Auranofin prevented the activation of IKK kinases and the degradation of IκBα (Figures 2B, 2C, and 2D). Auranofin also inhibits TNF- and IL-1-induced NEMO ubiquitination and IL-1-induced IRAK1 ubiquitination, raising the hypothesis that the molecule could act at the ubiquitination machinery level and even at upstream steps of the IKK activation (Figure 2E). The recruitment of NEMO to the TNF-R1 signaling complex (TNF-RSC) is dependent on its ubiquitination by the linear ubiquitination complex LUBAC (Haas et al., 2009). To monitor this recruitment, we stimulated U2OS cells with TNF-α for the indicated time and then immunoprecipitated the TNF-RSC and analyzed its interaction with NEMO. This experiment revealed that whereas NEMO and its ubiquitinated form were detected transiently after 5 min of stimulation with TNF-α, their interactions with TNF-RSC were strongly reduced upon Auranofin treatment (Figure 2F). Altogether, these experiments demonstrate that Auranofin acts upstream of NEMO ubiquitination to inhibit its recruitment to macromolecular complexes and to prevent activation of the IKK kinases, the core element of the NF-κB cascade.

### Effects of Auranofin on SARS-CoV-2-mediated NF-κB pathway and ISGylation

As Auranofin was shown to prevent SARS-CoV-2 replication (Biji et al., 2021; Rothan et al., 2020), we evaluated the effect of Auranofin on NF-κB pathway 48 h following SARS-CoV-2 infection (MOI 0.05) in Calu-3 cells (epithelial cells from a lung adenocarcinoma). As shown in Figure 2G, viral infection of Calu-3 increased IκBα and p65 phosphorylations, two hallmarks of NF-κB activation that are inhibited by Auranofin. We then decided to study in more detail the mechanism by which Auranofin affects SARS-CoV-2 infectivity. Having shown that Auranofin inhibits NF-κB activation in response to proinflammatory cytokines and SARS-CoV-2, we examined its effect on the IFN pathway, which is both regulated by the NF-κB and IRF3/IRF7 signaling pathways. Type I interferon is the first line of defense against viral infection through the induction of interferon-stimulated genes (ISGs). Among these ISGs, ubiquitin-like protein ISG15 is one of the most rapidly induced (Perng and Lenschow, 2018). ISG15 can be covalently conjugated to its targets through a process called ISGylation. Interestingly, the papain-like protease PLpro is an essential coronavirus enzyme required for processing viral polyproteins and it was shown recently that SARS-CoV-2-PLpro preferentially cleaves ISGylation of substrates (Klemm et al., 2020; Shin et al., 2020). Interestingly, gold metallodrugs including Auranofin are efficient inhibitors of PLpro of SARS-CoV-2 PLpro (Gil-Moles et al., 2020) and we suspect that it could restore ISGylations. However, as Auranofin is also an inhibitor of NF-κB and SARS-CoV-2 entry, it dampens the antiviral response and consequently the level of ISGylations (Figure S4).

### *In vitro* antiviral activity assays of NF-κB inhibitors on SARS-CoV-2-infected cells

**Figure 3:**
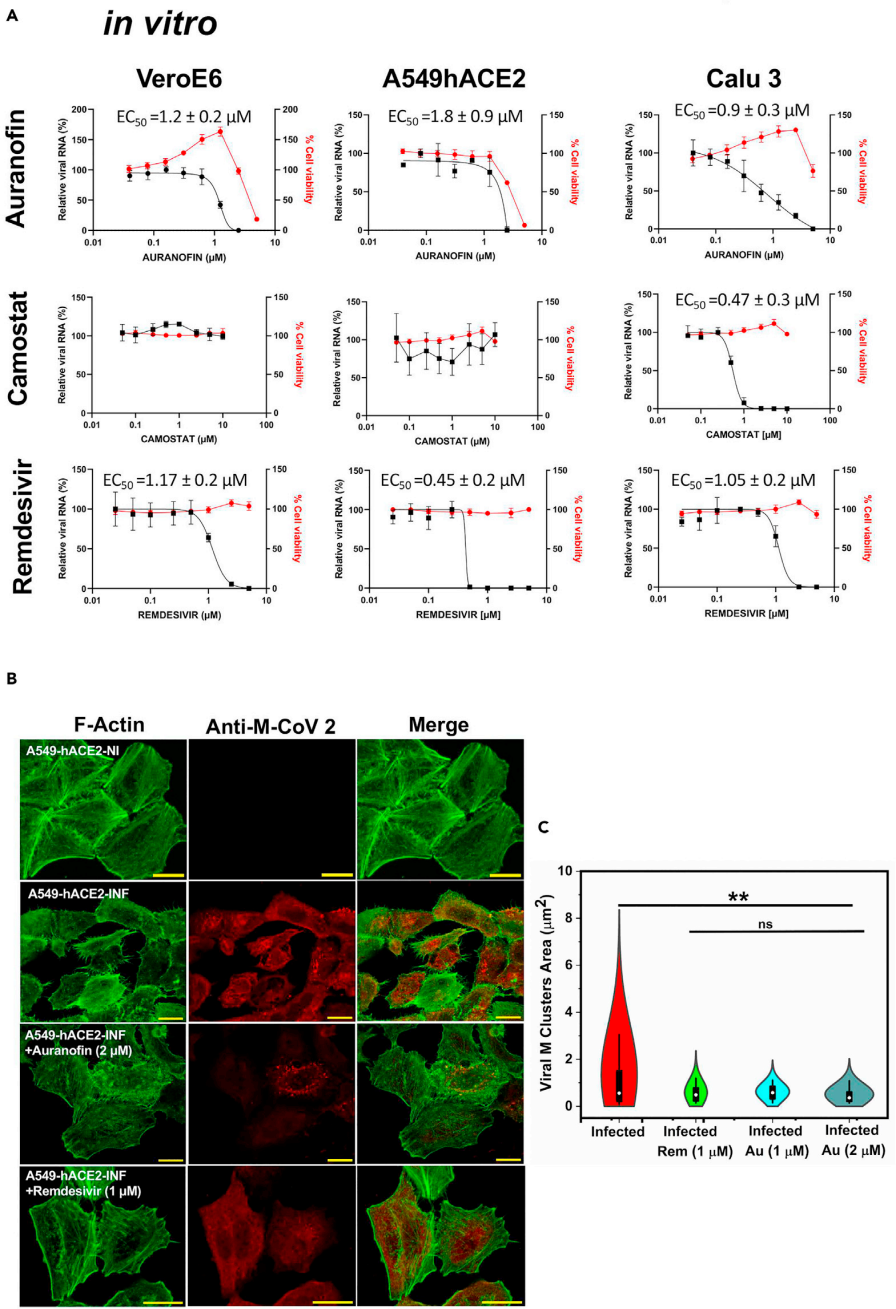
Effect of Auranofin on SARS-CoV-2 replication *in vitro* (A) Dose-dependent effect of Auranofin, Camostat or Remdesivir on simian Vero E6 cells, human pulmonary Calu-3 or A549-hACE2 cell lines. Cell viability was evaluated using MTS in all cell lines (red line). Cell lysates harvested 48 h post-infection (MOI of 0.01) were used for RT-qPCR on the SARS-CoV-2 E gene (Black line) to evaluate the antiviral activity (*n* = 3 independent experiments in duplicate). (B) Immunofluorescence of non-infected (NI) or SARS-CoV-2 infected (INF) A549-hACE2 cells in the absence or in the presence of Auranofin 2 μM or Remdesivir 1 μM. 48 h post-infection, cells were fixed, permeabilized and stained for anti-M SARS-CoV-2 (in red) and F-actin (in green) and imaged by fluorescent confocal microscopy. Scale bars, 17 μm. (C) SARS-CoV-2 M-labeled clusters inside cells or at the cell membrane were quantified for their size (area in μm^2^) and reported in the graph for each condition (*n* = 30–50 cells) showing a decrease in intracellular SARS-CoV-2 assembly clusters upon treatment with 1 or 2 μM of Auranofin, or 1 μM of Remdesivir as a control. ns: non-significant, ∗∗p<0,01. See also Figures S5 and S6.

We next sought to determine if Auranofin and Parthenolide, which were identified as inhibitors of NF-κB activation (Table 1) could protect cells from SARS-CoV-2 infection. First, we evaluated cell toxicity and viral RNA production on Vero E6, an African green monkey kidney-derived cell line, which is widely used as the infection model for SARS-CoV-2 and expresses ACE2 receptor and TMPRSS2 co-receptor as shown in Figure S5. Vero E6 cells were pretreated for 2 h with serial dilutions of the drugs, infected with SARS-CoV-2, and incubated with these compounds continuously for 48 h. Results showed that Auranofin presented an inhibitory effect on SARS-CoV-2 replication with an EC50 of 1.2 ± 0.2 μM, whereas Parthenolide and ML120B (a known NF-κB inhibitor) did not (Figure S6). We next compared the effect of Auranofin with that of other well-known drugs inhibiting TMPRSS2 (Camostat Mesylate) or RNA-dependent RNA polymerase (Remdesivir) in four different cell types (Figure 3A) including Vero E6 (described above), A549 (a selective clone of human alveolar pulmonary cells), A549-hACE2 overexpressing the receptor hACE2 and the pulmonary Calu-3 cells naturally expressing endogenous ACE2 and TMPRS22 (as shown on Figure S5). These cells were infected with SARS-CoV-2 and viral RNAs were isolated from cell extracts 48 h post-infection and quantified by RT-qPCR against the SARS-CoV-2 E gene. Cell viability was measured with the MTS assay. It is worth noticing that Auranofin increased cell viability in Vero E6 and Calu-3 cells in the micromolar range, before showing toxicity above 2.5 μM. Auranofin displayed a strong dose-dependent antiviral activity against SARS-CoV-2, similar to the one observed with Remdesivir (EC50 1.17 ± 0.2 μM) (Figure 3A). The EC_50 A549-hACE2_ for Auranofin was 1.8 ± 0.9 μM, slightly higher than the EC_50__Vero E6_ probably owing to the over-expression of hACE2 in these cells. In Calu-3 cells, we observed that Auranofin was responsible for a strong dose-dependent reduction of SARS-CoV-2 replication with an EC_50_ of 0,9 ± 0,35 μM, in the same range as the Remdesivir control (EC_50_ of 0.9 ± 0,3 μM) but 2-fold higher than Camostat Mesylate (EC50 of 0.47 ± 0.3 μM). Interestingly, Camostat showed a dose-dependent effect only in Calu-3 cells, as Vero E6 and A549-hACE2 present very low levels of active TMPRSS2 (David et al., 2022; Wilson et al., 2005). Together, these results show that Auranofin exhibits an inhibitory effect against SARS-CoV-2 infection in relevant human pulmonary cell lines, most probably targeting virus entry through ACE2-dependent endocytosis and not through the TMPRSS2 pathway.

To get further insights into the mechanism of action of Auranofin, we monitored SARS-CoV-2 infection in Auranofin-treated A549-hACE2 cells by confocal microscopy using fluorescently labeled antibodies against M protein (Figure 3B), one of the main structural transmembrane glycoprotein of SARS-CoV-2 required for particle assembly. Quantification of M cluster area per cell showed a 6-fold decrease of intracellular M clusters in the presence of Auranofin (1 or 2 μM) as compared with non-treated cells (Figure 3C). These results suggest that Auranofin induces a blockage of viral particles at the cell membrane upon entry. This pattern is different from that of Remdesivir-treated cells where only a reduction of intracellular viral M clusters was observed without any virus plasma membrane labeling (Figures 3B and 3C, Remdesivir) in agreement with data showing that Remdesivir is targeting the viral replication complexes before M clusterization for assembly (Bracquemond and Muriaux, 2021). These data suggest that independently of its NF-κB inhibitory effect, Auranofin could act directly or indirectly on SARS-CoV-2 receptor or co-receptor or could affect the structural organization of the plasma membrane impacting viral membrane fusion or virus endocytosis.

### Auranofin affects the localization of Caveolin-1 but not ACE2, NRP1, and TMPRSS2 in lipid microdomains

Lipid microdomains have been suggested to be important for SARS-CoV-2 infection, using pseudoviruses and HEK cells expressing ACE2 (Li et al., 2021). Indeed, some studies propose that they affect the localization of the receptors and coreceptors of the virus (Glende et al., 2008; Lu et al., 2008), whereas others suggest a role for their composition in cholesterol (Palacios-Rápalo et al., 2021).

**Figure 4:**
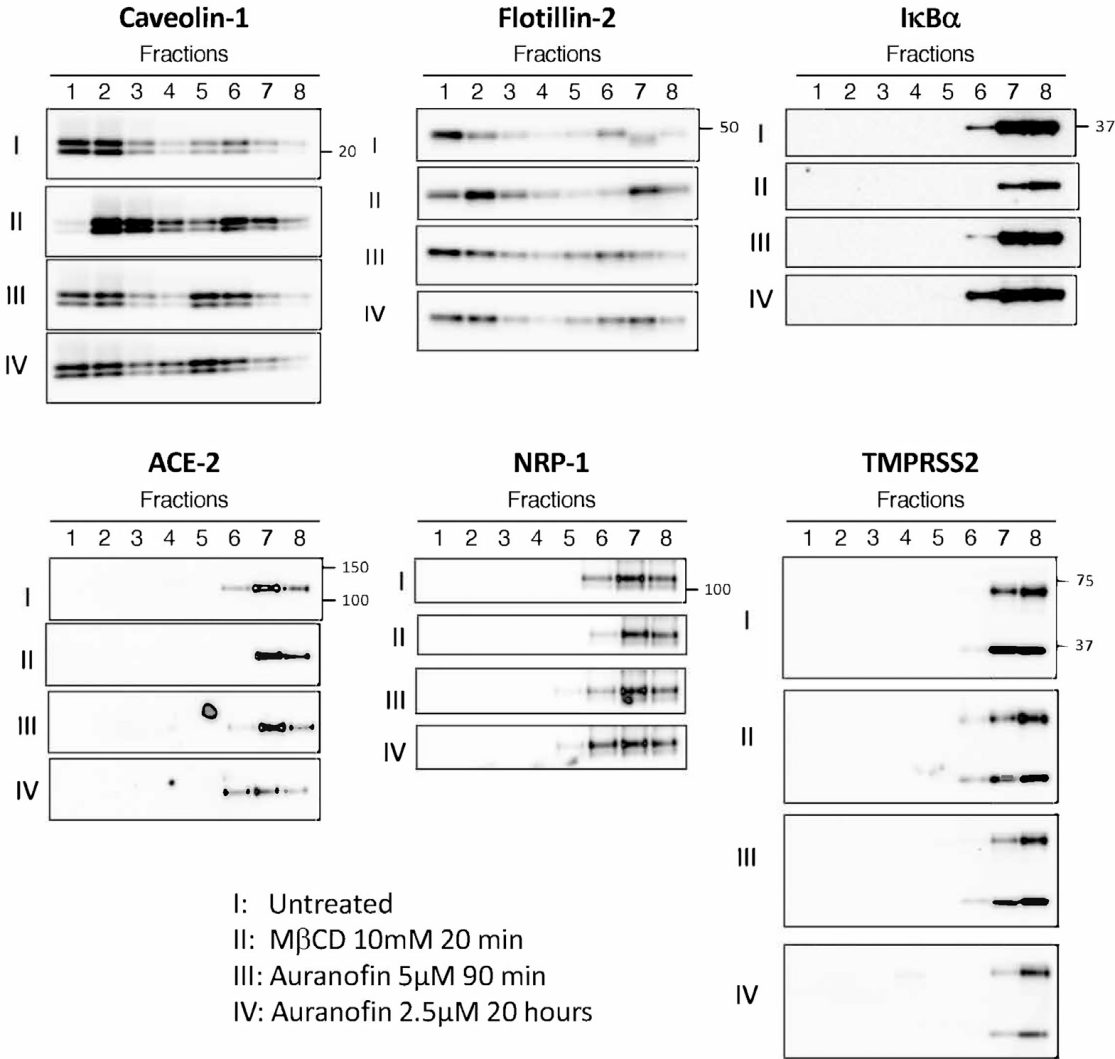
Effect of Auranofin on lipid rafts-resident proteins Sucrose gradient fractionation of A549-hACE2 cell extracts following treatment with MβCD or Auranofin at the indicated times and concentrations. Individual fractions were analyzed by immunoblot using antibodies against Caveolin-1, Flotillin-2, ACE-2, TMPRSS2, NRP-1, and IκBα. See also Figures S7 and S8.

To determine the impact of Auranofin on the membrane localization of SARS-CoV-2 receptors and coreceptors, sucrose gradient flotation assays were performed (Figure 4). As expected, in untreated conditions, the raft resident proteins Caveolin-1 and Flotillin-2 were mainly localized in the low-sucrose detergent-resistant fractions (fractions 1–4). Interestingly, short-term (90 min at 5μM) or long-term (20 h at 2.5μM) treatment with Auranofin led to a redistribution of Caveolin-1 and Flotillin-2 from low-to high-density fractions, similar to what was observed with cholesterol depletion induced by Methyl−β-cyclodextrin (MβCD) (Figure 4). However, we did not detect SARS-CoV-2 receptors and coreceptors, ACE2, TMPRSS2 nor NRP-1, in the low-density fractions, whether or not the cells were treated with Auranofin or MβCD (Figure 4).

To determine whether the effect of Auranofin on Caveolin-1 and Flotillin-2 localization was owing to changes in cholesterol content and/or membrane organization, we first measured the amount of cholesterol in cell membranes using Amplex Red. In contrast to MβCD, Auranofin did not decrease the amount of cholesterol in isolated membrane fractions from Vero E6 cells (Figure S7A). To measure the direct effect of Auranofin on cellular cholesterol, we loaded Vero E6 or A549-hACE2 cells with H^3^ cholesterol (Figure S7B). As expected, MβCD led to an extraction of cholesterol from the plasma membrane, whereas no effect of Auranofin on the level of incorporated H^3^ cholesterol was observed after 4 or 24 h of treatment, indicating that the mechanism of action of Auranofin on cholesterol, if any, would be different from that of MβCD. As shown in Figure S7C, cholesterol efflux evaluated by the addition of human serum was not affected by Auranofin, indicating it does not affect the activity of cholesterol transporters. Although it was reported that MβCD affects infectivity of SARS-CoV-2 pseudoviruses in HEK293T-ACE2^hi^ cells (Li et al., 2021), we did not observe any effect of MβCD on SARS-CoV-2 infection in Vero E6 cells, arguing against a critical role of membrane cholesterol in SARS-CoV-2 infectivity (Figure S6).

We then used the Nile Red-derivative NR12S probe (Kucherak et al., 2010), a compound that once incorporated into the plasma membrane allows us to monitor the lipid ordering of this membrane. Indeed, the fluorescence emission spectra of NR12S change in response to lipid order, shifting toward shorter wavelengths when incorporated into a liquid-ordered phase compared with a liquid-disordered phase. We observed that similarly to MβCD, Auranofin treatment reduced in a dose-dependent manner the ratio of the fluorescence emission intensities at 580/630nm, indicating a change in lipid order of the plasma membrane upon Auranofin treatment (Figure S8) (Saxena et al., 2014). Altogether, our results demonstrate that Auranofin is responsible for the redistribution of lipid microdomains resident proteins without affecting the amount of cholesterol at the cell surface.

### Auranofin affects the diffusion of ACE2 receptors at the cell plasma membrane

**Figure 5:**
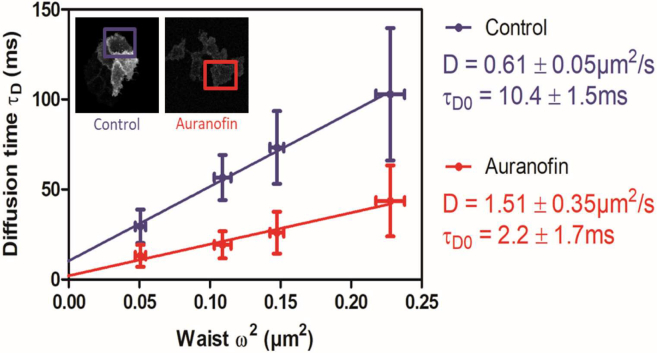
Auranofin increases hACE2^mScarlet-1^ mobility at the membrane of pulmonary cells Images of A549-hACE2^mScarlet-1^ human pulmonary cells at 37°C for 1 h with Auranofin (5 μM) or without (control) as indicated. Measurement of hACE2^mScarlet-1^_1_ receptor mobility (D, diffusion) at the cell surface of pulmonary cells after 1-h treatment with Auranofin (red line) or without Auranofin (control, blue line) using svFCS.

We used Fluorescence correlation spectroscopy (FCS) to measure the effect of Auranofin on ACE2 dynamics at the cell membrane of A549 pulmonary cells. This technique, in which the temporal resolution is improved without any loss of information, allowed us to identify micrometer-scale domains present in the plasma membrane (Favard et al., 2019). This approach assumes that if ACE2^mScarlet-1^ diffuses freely, one can expect to obtain a linear function between t_d_ and w^2^, the slope corresponding to the diffusion coefficient D in this environment. We first measure the diffusion coefficient of ACE2^mScarlet-1^ applying the FCS diffusion law giving the diffusion time versus the beam area. We found that Auranofin increases the diffusion coefficient of ACE2 by 3-fold (Figure 5), suggesting that Auranofin could prevent optimal receptor recognition by the virus by increasing ACE2 receptor mobility in the cell membrane.

### Auranofin does not prevent syncytia formation

We examined whether Auranofin could inhibit syncytia formation using a GFP-split complementation system in which two HEK 293T populations separately produce half of the reporter protein, namely GFP1-10 and GFP11, which becomes fluorescent upon cell–cell fusion (Buchrieser et al., 2020; Sanders et al., 2021). HEK 293T ACE2-GFP-split were cocultured with HEK 293T S-GFP-split at different cell densities in the presence of increasing concentrations of Auranofin (0.1 nM–10 μM) and spontaneous syncytia formation was quantified 48 h post-treatment with the Opera Phenix high-content screening system (Figure S9). Auranofin did not prevent cell–cell fusion when non-toxic concentrations were used, indicating that Auranofin does not counteract cell fusion and syncytia formation that is triggered by the interaction of Spike with ACE2 (Buchrieser et al., 2020; Sanders et al., 2021).

### Auranofin inhibits the raft-dependent endocytic pathway responsible for SARS-CoV-2 cell entry

**Figure 6:**
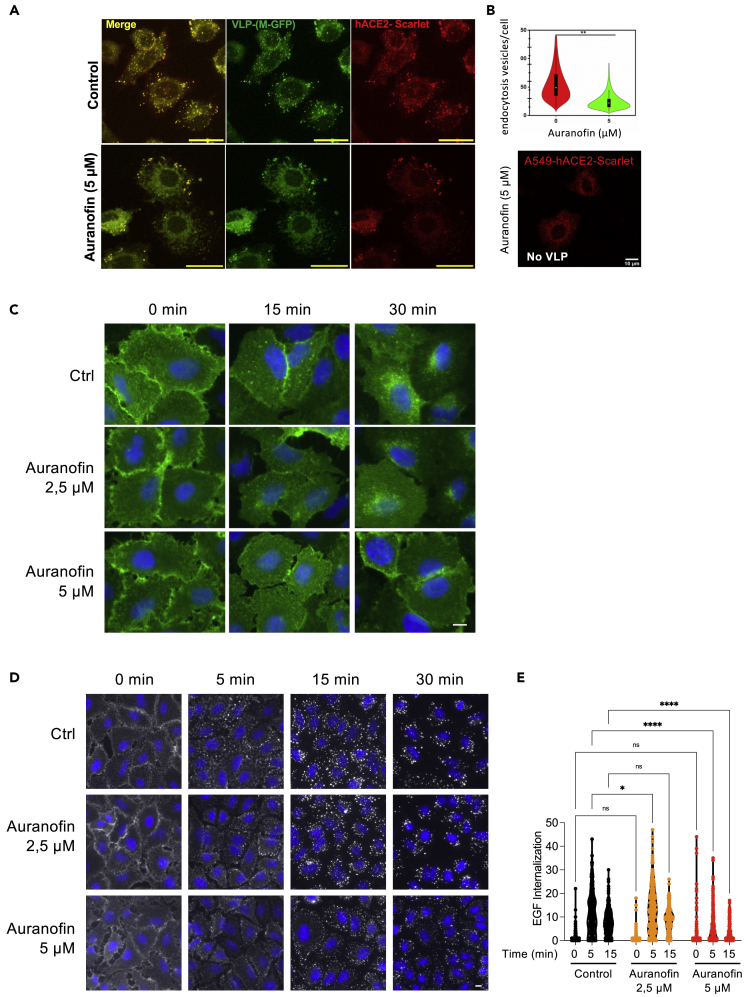
Auranofin inhibits endocytosis (A) Auranofin inhibits SARS-CoV-2 VLP(GFP)-Spike internalization in A549-hACE2^mScarlet-1^ pulmonary cells. MNES-VLP(M-GFP) were incubated on A549-hACE2^mScarlet-1^ human pulmonary cells at 37°C for 1 h with or without Auranofin (5 μM). Panel A shows Z-projection images of MNES-VLP(GFP) and ACE2^mScarlet-1^ for VLP internalization visualization at 37°C without Auranofin (control) or with Auranofin (5 μM for 1 h). Scale bar, 10 μm. (B) Plot for the number of endocytosis vesicles per cell (*y*-axis) without Auranofin (control) or with Auranofin (5 μM) (*x*-axis). A number of 20 < *n* < 40 cells were analyzed from at least three independent experiments. Statistical analyses were evaluated using one-way ANOVA and *t*-tests. *p* < 0.05 is significant. Example of a confocal microscopy image showing hACE2^mScarlet-1^ receptor distribution in cells without any VLP (No VLP). (C) Auranofin inhibits raft-dependent endocytosis. A549-hACE2 cells pretreated with Auranofin at the indicated concentrations were labeled with FITC-coupked cholera toxin B subunit for 1 h at 4°C and then chased in serum-free medium for 15 or 30 min at 37°C, a condition allowing its endocytosis. Cells were then fixed in 4% paraformaldehyde, stained with DAPI and analyzed by microscopy. Scale bar, 5 μm. (D) Auranofin inhibits EGF endocytosis. A549-hACE2 cells treated or not with Auranofin at the indicated concentrations for 2 h were stimulated with Alexa Fluor 555-coupled EGF for 5, 15, or 30 min at 37°C. Cells were then fixed in 4% paraformaldehyde, stained with DAPI and analyzed by microscopy. Scale bar, 5 μm. (E) Quantification of EGF internalization (related to Figure 6D). Statistical significance was determined by ANOVA with Bonferroni post hoc correction for multiple comparisons; ns, non-significant. ns: non-significant, ∗p<0,05, ∗∗∗∗p<0,0001. See also Figure S10.

As we observed an accumulation of virus particles at the cell membrane following Auranofin treatment (Figure 3B), we next assessed the effect of Auranofin on viral entry by endocytosis. Fluorescently labeled SARS-CoV-2 virus-like particles (VLP) harboring the Spike with green fluorescence M protein (MNES-(M)GFP VLP) were generated and used to investigate the effect of Auranofin on SARS-CoV-2 entry via endosomal entry pathway using A459-hACE2^mScarlet-1^ cells that express a fluorescent-tagged version of ACE2 (Figure 6A) (Hoffmann et al., 2020; Zhu et al., 2021). In the absence of Auranofin, a dispersed localization of VLP(GFP)-Spike was observed in the A459-h ACE2^mScarlet-1^ cells (control), indicative of VLP internalization following recognition of VLP-Spike by ACE2, as confirmed by a strong colocalization between the two signals (Figure 6A, merge). In contrast, the VLP(GFP)-Spike was mainly detected at the cell periphery where it also colocalized with ACE2^mScarlet-1^_1_ in 5 μM Auranofin-treated cells albeit not entering the cells and staying blocked either at the cell plasma membrane or the early steps of endocytosis. Quantification data analysis of the number of VLP(GFP) per cell in the z-projection images reveals an average (mean per cell) of 57 ± 28 endocytosed VLP in the absence of Auranofin and 25 ± 13 in the presence of 5 μM Auranofin, showing a significative difference (p < 0.05) with a 2-fold decrease of VLP entry upon Auranofin treatment (Figure 6B). This striking reduction of VLP-Spike-ACE2 internalization upon Auranofin treatment suggests that Auranofin inhibits ACE2-dependent endosomal viral entry without preventing VLP-Spike-ACE2 association. Interestingly, we noticed that without VLP, the ACE2m^mScarlet-1^ receptor is not organized in dots (see the image of ACE2^mScarlet-1^ cells “no VLP”). We next addressed the issue of the type of endocytic pathway (clathrin-dependent or independent) affected by Auranofin. Effect of Auranofin was assessed on the internalization of (1) Cholera toxin, endocytosed in a raft-dependent manner, (2) Epidermal growth factor receptor (EGFR), endocytosed via clathrin-dependent and independent endocytosis processes, and, finally, (3) Transferrin receptor, internalized by clathrin coated-pits.

Cells were incubated for 1 h at 4°C with Cholera toxin B subunit (CTxB), which binds to the lipid-raft resident glycosphingolipid GM1 (Kenworthy et al., 2021; Sethi et al., 2019), and then chased in serum-free medium for 30 min at 37°C, a condition allowing its endocytosis. Incubation of cells with 5 μM Auranofin, but not with 2.5 μM, inhibited the accumulation of CTxB into endosomal structures (Figure 6C). Analogously, 5 μM Auranofin treatment strongly reduced the EGFR internalization (Figures 6D and 6E). In contrast, neither 2.5 nor 5 μM of Auranofin inhibited Transferrin internalization indicating that Auranofin does not prevent clathrin-mediated endocytosis (Figure S10).

Altogether, our results suggest that Auranofin prevents VLP endocytosis by a raft-dependent rather than by a clathrin-dependent endocytosis mechanism.

## Discussion

The originality of a drug screening relies on the target choice and the assay method. The novelty of our screening method was to search for inhibitors of NF-κB activation events occurring early time of induction and upstream of the IKKs activation and the transcriptional activation mediated by NF-κB. Previously published screens used conventional methods such as NF-κB reporter genes assays to test various chemical libraries in cancer cells or DNA strand exchange fluorescence resonance energy transfer to identify p65/RelA-specific inhibitors (Baudy et al., 2009; Shiroma et al., 2020). Therefore, these screens identified several new NF-κB inhibitors, but none of those were identified in our screen. We found 60 compounds among which we selected four hits: Phenoxybenzamine, Thimerosal, Parthenolide`, and Auranofin, able to inhibit not only NF-κB-dependent transcription but also the formation of the NF-κB activating complexes. In this short list, only Auranofin and Parthenolide present few side effects and have been reported to interfere with NF-κB through their ability to inhibit IKK kinases (Jeon et al., 2003; Saadane et al., 2007). Phenoxybenzamine is an α-adrenergic blocker used to treat high blood pressure and pheochromocytoma. The adverse effects of this treatment are mostly orthostatic hypotension, tachycardia, dizziness, drowsiness, and stomach upset. Thimerosal is an organomercurial used as a preservative for certain vaccines. This compound is however too toxic to be used therapeutically. Parthenolide is a sesquiterpene lactone derived from the medicinal herb feverfew that has been shown to have anti-inflammatory and anti-cancer properties (Wang and Li, 2015). It inhibits the expression of COX-2 and proinflammatory cytokines (TNF-α and IL-1). Parthenolide was also shown to inhibit DNMT1, a DNA methyltransferase whose expression is also decreased by SARS-CoV-2 infection, leading to promoter hypomethylation (Liu et al., 2009; Muhammad et al., 2021). Auranofin is an anti-inflammatory gold salt used previously to treat rheumatoid arthritis. We focused on this molecule because it has garnered renewed attention in recent years owing to its therapeutic potential for various pathologies including cancer, infectious diseases, and neurodegenerative diseases (Balfourier et al., 2020). We demonstrated that Auranofin is a potent inhibitor of NF-κB in response to proinflammatory cytokines (TNF-α, IL-1), LPS, and SARS-CoV-2 infection. We found that in contrast to the IKK inhibitor ML120B, Auranofin prevents the formation of activation complexes containing NEMO, suggesting that Auranofin acts upstream of IKK kinase activation. Consistently, we found that Auranofin inhibits IL-1- or TNF-α-induced ubiquitination of NEMO, a signaling event that occurs rapidly after exposure of cells to inflammatory cytokines and is required for the recruitment of NF-κB effectors to TNF-RSC. However, it is still unclear which proteins Auranofin targets in the NF-κB pathway. Interestingly, proteomic approaches have recently identified NFKB2 (Nuclear Factor kappaB p100 subunit), CHORDC1 (Cysteine and Histidine-Rich Domain-Containing 1), and TXNRD1 (Thioredoxin Reductase 1) as the main targets of Auranofin (Saei et al., 2020). Similarly, Auranofin was also shown to inhibit the proteasome-associated deubiquitinases UCHL5 (Ubiquitin C-Terminal Hydrolase L5) and USP14 (Ubiquitin Specific Peptidase 14) which could also be responsible for the observed effects (Zhang et al., 2019). Further studies are required to determine the role of these targets in Auranofin-mediated inhibition of NF-κB by assessing, for example, the effect of their depletion on NF-κB activation complexes.

The discovery of inhibitors of the NF-κB pathway is of major interest for various viral infections and pathologies such as cancers and autoimmune diseases, which are often associated with constitutive activation of NF-κB responsible for the expression of anti-apoptotic genes and the production of proinflammatory cytokines responsible for pathogenic lesions. In the current context, we have decided to focus on COVID-19, a disease whose severity relies on uncontrolled cytokine response, a process regulated by NF-κB. Among our candidates, we sought to identify which ones could inhibit SARS-CoV-2 infection in addition to preventing NF-κB activation. Four recent publications have pointed Auranofin as a potential treatment for COVID-19 (Biji et al., 2021; Cirri et al., 2021; Rothan et al., 2020; Ullrich and Nitsche, 2020). However, these studies do not clearly assign the mechanism(s) by which Auranofin affects SARS-CoV-2 infection. Accordingly, we confirmed that Auranofin could decrease the infection by SARS-CoV-2 in more relevant cell lines, i.e., human alveolar pulmonary cells. Remarkably, we have shown that Auranofin blocks viral entry into cells and as a result decreases the number of replication complexes. Mechanistically, we showed that Auranofin does not affect ACE2 expression and localization at the plasma membrane, nor that of its other coreceptors NRP1 and TMPRSS2, although it modifies the localization of the lipid raft markers Caveolin-1 and Flotillin-2. In fact, we observed that ACE2 is not present in lipid rafts at a steady state as previously reported (Li et al., 2007; Parkin et al., 2003; Warner et al., 2005) but in contrast to two other reports (Glende et al., 2008; Lu et al., 2008). We cannot exclude however that SARS-CoV-2 receptors and coreceptors are only weakly associated with rafts and dissociated from this compartment in the extraction procedure. Alternatively, they may localize in the lipid-raft compartment only after SARS-Cov2 infection.

Our results show that Auranofin increases significantly ACE2 membrane diffusion, which depends on membrane components (including cholesterol), cytoskeleton, or ACE2 modifications such as those mediated by thioredoxin (Singh et al., 2020). We thus explored the effect of Auranofin on the interplay between SARS-CoV-2 receptors/coreceptors and membrane cholesterol. We did not observe any effect of Auranofin on the release of cholesterol, nor alteration of ACE2/TMRPSS2/NRP1 sedimentation properties following depletion of cholesterol by MβCD. Moreover, unlike Auranofin, MβCD did not affect SARS-CoV-2 infectivity. Using SARS-CoV-2 pseudo-particles, we found that Auranofin could efficiently inhibit their endocytosis, a cellular process involved in SARS-CoV-2 cellular entry (Bayati et al., 2021), by interfering with raft-dependent rather than clathrin-dependent endocytosis mechanism.

**Figure 7:**
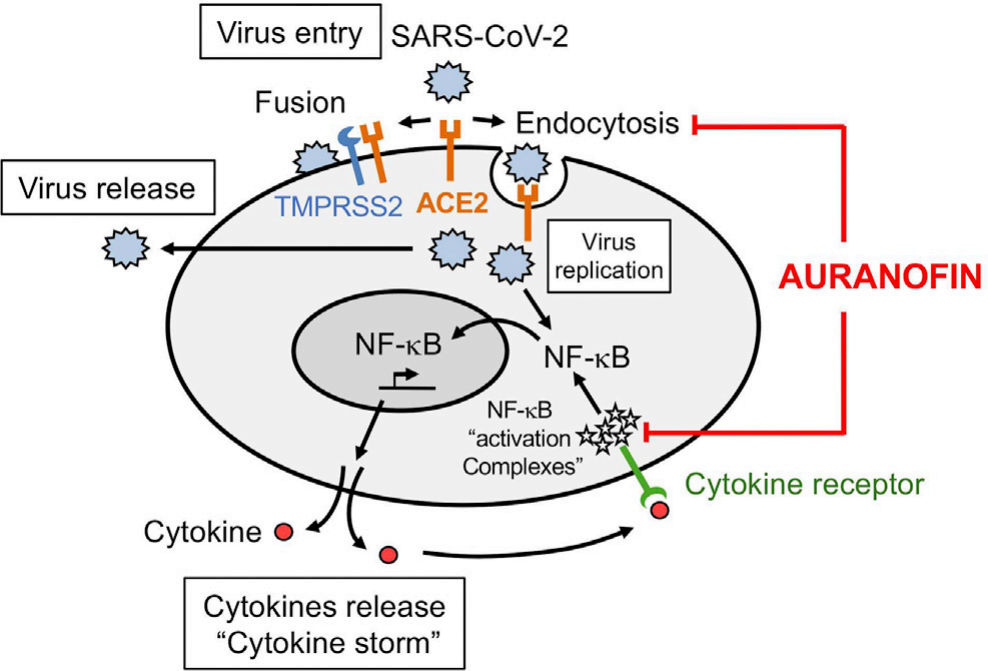
Schematic model showing the dual roles of Auranofin in countering SARS-CoV-2 infection and inflammatory cascades We propose a model in which Auranofin acts on the diffusion of ACE2, the main receptor of SARS-CoV-2, and lipid microdomains with the consequence of a significant effect on endocytosis, which is one of the mechanisms of viral entry. Our data show that Auranofin targets early events of the NF-κB signaling pathway and inhibits its activation both in response to viral entry and the cytokine hyperproduction that correlates with multi-organ systemic complications, eventually leading to death.

We thus propose a model by which Auranofin could impact different cellular processes and counter SARS-CoV-2 infection and its pathogenicity (Figure 7). We think that Auranofin disrupts the organization of membrane lipids, an event that could impact many cellular signaling pathways including NF-κB, endocytosis, and ACE2 receptor diffusion (Legler et al., 2003). Given the importance of membrane fusion in SARS-CoV-2 infectivity, we anticipated that Auranofin could impact this cellular event. However, GFP-split complementation experiments indicate that Auranofin does not affect syncytia formation, suggesting that it affects another mechanism responsible for viral entry and we showed that it targets raft-dependent endocytosis.

Interestingly, genome-wide CRISPR screens and pharmacological studies have recently revealed that cholesterol homeostasis is critical for SARS-CoV-2 entry (Daniloski et al., 2021; Wang et al., 2021; Wei et al., 2021). Indeed, alterations of genes that control the transcription of lipogenic enzyme synthesis (such as the transcription factor SEBP), the ER to Golgi trafficking of SEBP (such as INSIG/SCAP), the cellular entry or trafficking of cholesterol (LDLR, CCC protein complex, Rab7) impair SARS-CoV-2 entry. After endocytosis, LDL is delivered to the lysosomes, where cholesterol esters are cleaved by acid lipase. Mutation of the proteins NPC1 and NPC2 leads to the Niemann-Pick syndrome disease as a result of an accumulation of cholesterol in the lysosomes. These alterations of cholesterol disturb lysosome functions that are required for Spike modifications by Cathepsin L and B, therefore affecting viral fusion (Ballout et al., 2020). In addition, compounds that regulate SEBP trafficking/activity or block lipids peroxidation inhibit SARS-CoV-2 infection (Schmidt et al., 2020). The steps of these processes that could be affected by Auranofin are under current evaluation. A primary target of Auranofin is the 3-hydroxy-3-methylglutaryl-coenzyme A reductase (HMGCR) that regulates the cholesterol biosynthesis pathway, also referred to as the mevalonate cascade (Tian et al., 2019). However, it is unlikely that this target is responsible for the disturbances in the membrane mobility of cholesterol, which occurs rapidly.

In summary, using model cell lines we show that Auranofin acts at several levels to interfere with SARS-CoV-2 entry by endocytosis and through the inflammatory response, which is a source of morbidity in COVID-19. We thus propose that Auranofin could be an attractive therapeutic option to improve severe forms of COVID-19. *In vivo* studies using animal models as well as clinical studies would make it possible to validate the therapeutic potential of Auranofin.

### Limitations of the study

Experiments of infection with SARS-CoV-2 presented in this work were performed using a single round of infection (over 72 h) and not over several days/weeks using a multi-step growth curve analysis. Such experiments would allow for the assessment of a possible SARS-CoV-2 escape from the drug over time.

Our work identifies Auranofin as a potential therapeutic drug for the treatment of COVID-19. Our data were obtained in cell lines that represent good models for SARS-CoV-2 biology. More physiological settings such as 3D pulmonary organoid cultures or animal models (K18-ACE2 mice, SARS-CoV-2-adapted mice, or hamsters) would make it possible to define the real therapeutic potential of Auranofin. Studies in human cohorts would finally demonstrate the benefit of Auranofin treatment.

## STAR★Methods

### Key resources table

**Table:** 

REAGENT or RESOURCE	SOURCE	IDENTIFIER
**Antibodies**		

Anti-NEMO	Santa Cruz Biotechnology Inc	Cat# sc8330; RRID: AB_2124846
Anti-NEMO	Becton Dickinson	Cat#611306; RRID: AB_2124846
Anti-IRAK1	Santa Cruz Biotechnology Inc	Cat#sc7883; RRID: AB_2233753
Anti-IRAK1	Santa Cruz Biotechnology Inc	Cat#sc5286; RRID: AB_628411
Anti-IKKβ	Abgent	Cat#8109a
Anti phospho-IKKα/β (Ser176/180)	Cell Signaling Technology	Cat#16A6; RRID: AB_2079382
Anti-IκBα	Becton Dickinson	Cat#610690
Anti-tubulin β	Sigma-Aldrich	Cat#T4026; RRID: AB_477577
Anti-Sharpin	Proteintech	Cat#14626-1-AP; RRID: AB_2187734
Anti-Caveolin-1	Becton Dickinson	Cat#610406; RRID: AB_397788
Anti-ACE2	R&D	Cat#AF3437; RRID: AB_223140
Anti-ACE2	Santa Cruz Biotechnology Inc	Cat#E11; RRID: AB_2861379
Anti-NRP1	Proteintech	Cat#60067-1-Ig; RRID: AB_2150840
Anti-TMPRSS2	Santa Cruz Biotechnology Inc	Cat#H4
Anti-ISG15	Abgent	Cat#AP1150a
Anti-phospho-STAT1 (Y701)	Cell Signaling Technology	Cat#9167; RRID: AB_561284
Anti-IRF7	Santa Cruz Biotechnology Inc	Cat#sc9083
Anti-Flag	Merck	Cat#M2; RRID: AB_262044
Anti-Flotillin-2	Becton Dickinson	Cat#610383; RRID: AB_397766
Anti-P65	A gift fron Nancy Rice	Cat#1226
Anti-IRAK1	Santa Cruz Biotechnology Inc	Cat#F4
Anti-Mouse IgG (H + L) HRP	Jackson ImmunoResearch	Cat#31430; RRID: AB_228307
Anti-Rabbit IgG (H + L) HRP	Jackson ImmunoResearch	Cat#111-035-144
Anti-Goat IgG (H + L) HRP	Jackson ImmunoResearch	Cat#705-035-003; RRID: AB_2340390
Anti-Mouse Alexa Fluor 488	Thermofischer Scientific	Cat#A32723; RRID: AB_2633275
Anti-Rabbit Alexa Fluor 488	Thermofischer Scientific	Cat#A32731; RRID: AB_10003985
Anti-Goat Alexa Fluor 488	Thermofischer Scientific	Cat#A32814; RRID: AB_2762838
Anti-Rabbit IgG Alexa Fluor 555	Life Technologies	Cat#A-21428; RRID: AB_141784
Anti-TNFR1-coupled to PE	Miltenyi	CD120a, Cat#REA252
Isotype control antibody coupled to PE	Miltenyi	Cat#
Goat anti-IL1-R1	R&D	Cat#AF269; RRID: AB_355286
Anti-M of SARS-CoV-2	TEBU-BIO	Cat#100-401-A55
Donkey anti-Rabbit AF568	Life Technologies	Cat#A10042; RRID: AB_2534017

**Virus strains**		

BetaCoV/France/IDF0372/2020	National Reference Center for Respiratory Viruses hosted by Institut Pasteur (Paris, France) and headed by Pr. Sylvie van der Werf	provided by Dr. X. Lescure and Pr. Y. Yazdanpanah and supplied through the European Virus Archive goes Global (EVAg) platform, a project that has received funding from the European Union’s Horizon 2020 research and innovation program under the grant agreement No 653316
SARS-CoV-2 virus-like particles		Swann, H. et al. Minimal system for assembly of SARS-CoV-2 virus like particles. *Sci Rep* 10, 21877 (2020).

**Chemicals, peptides, and recombinant proteins**		

Auranofin	Sigma Aldrich	Cat#A6733
Remdesivir	TEBU	Cat#282T7766
ML120B	Sigma Aldrich	Cat#783348-36-7
Camostat mesylate	Sigma Aldrich	Cat#SML0057
Parthenolid	Sigma Aldrich	Cat#P0667
MbCD	Sigma Aldrich	Cat#C4555
Fetal bovine serum	Gibco Life Technologies	Cat#11560636
Penicillin/Streptomycin	Gibco Life Technologies	Cat#11548876
Prestwick Chemicals	1,270 non-patent drugs fully approved by the FDA and other agencies	
Proteases inhibitor/complete	Merck	Cat#11697498001
Phosphatases inhibitor/PhosSTOP	Merck	Cat#4906845001
4–12% acrylamide precast gels	Biorad	Cat#3450124
PVDF membranes	Millipore	Cat#Immobilon#IPVH00010
Mowiol	Biovalley	Cat#17951-500
Phalloïdine-AF488	Life technologies	Cat#A12379

**Recombinant proteins**		

IL-1	Biotechne R&D system	Cat#201LB
TNF-α	Biotechne R&D system	Cat#210TA
TNF-α	AB biosciences	P-713M
Biotinylated-EGF/streptavidin Alexa 555	Thermo Fisher	Cat#E35350
Choleratoxin-FITC	Merck	Cat#C1655
Transferrin Alexa 488	Thermo Fisher	Cat#T13342

**Critical commercial assays**		

Bradford assay	Biorad	Cat#500006
Non-enzymatic dissociate buffer	Merck	Cat#C5914
MTS assay	Promega	Cat#G5421
Viral extraction with Cell Ready Lysis Module	New England Biolabs	Cat#E3032S
Alexa Fluor 488 Phalloidin (F-Actin labeling)	Thermo Fisher	Cat#A12379
Antifade Reagents	Tocris Bioscience	Cat#4055
Prolong GOLD antifade non DAPI	Invitrogen	Cat#P36934
Prolong GOLD antifade DAPI	Invitrogen	Cat#P36935
Amplex® Red cholesterol assay kit	Life Technologies	Cat##A12216
NR12S Supplier	Tocris Bioscience	Cat#1221739-85-0

**Experimental models: Cell lines**		

Vero E6 cells (African green monkey kidney cells)	ECACC	
A549 cells	ECACC	Cat#86012804
The human pulmonary alveolar A549-hACE2 were engineered using a lentiviral vector from Flash Therapeutics (Toulouse, France) to express the human receptor ACE2		
A549-hACE2scarlett that express a fluorescent tagged version of ACE2 were engineered		
Calu-3 cells (epithelial lung adenocarcinoma cells)	Elabscience	Cat#EP-CL-0054
U2OS	ATCC	Cat#HTB-96

**Oligonucleotides**		

SARS-CoV-2 E gene forward primer		
E_Sarbeco-F ACAGGTACGTTAA TAGTTAATAGCGT		
SARS-CoV-2 E gene reverse primer		
E_Sarbeco-R ATATTGCAGCAGTAC GCA CACA		
GADPH forward primer		
GCTCACTGGCATGGCCTTCCGTG		
GAPDH reverse primer		
TGGAGGAGTGGGTGTCGCTGTTG		

**Software and algorithms**		

Columbus Image data storage	PerkinElmer Technologies	https://www.perkinelmer.com/fr/product/image-data-storage-and-analysis-system-columbus
FlowJo 10.0 software	BD Biosciences	https://www.appliedcytometry.com/?gclid=EAIaIQobChMIrung-8SM-AIVLJBoCR09CwR9EAAYAiAAEgJRtPD_BwE
AxioVisio software	Zeiss	https://www.micro-shop.zeiss.com/en/us/system/software+axiovision-axiovision+program-axiovision+software/10221/
Zen software	Zeiss	https://www.zeiss.fr/microscopie/produits/microscope-software/zen.html
CellProfiler software		https://cellprofiler.org/
ImagJ/Fiji software	Fiji	https://imagej.net/software/fiji/downloads
Harmony software	Perkin Elmer	https://support.myharmony.com/en-us/download

**Statistical analysis**		

Origin 19 software	Origin lab	https://www.originlab.com/origin
One-way ANOVA		https://statistics.laerd.com/statistical-guides/one-way-anova-statistical-guide.php

**Other**		

Multi-Drop combi reagent dispenser	Thermo Fisher Scientific	Cat#5840300
T4 Cellometer cell counter	Nexcelom	
Multi-Channel Arm™ 384	TECAN	Cat#MCA 384
INCell 2000 automated wide-field system	GE Healthcare	
Nikon 20X/0.45, Plan Apo, CFI/60		
LSM780 laser-scanning microscopy	Zeiss	
Apotome imaging system	Zeiss	Imager Z1
Confocal microscope	Perkin Elmer	
Opera Phenix high-content screening system	Perkin Elmer	
Chemidoc touch imaging device	Biorad	
Glass Dounce homogenizer	Polylabo	
LSRFortessa™ Cell Analyzer	Becton-Dickinson Biosciences	
Fluorescence microplate reader	Molecular Devices	FexStation 3

### Resource availability

#### Lead contact

Further information and requests for resources and reagents should be directed to and will be fulfilled by the lead contacts, Robert Weil (robert.weil@upmc.fr) and Delphine Muriaux (delphine.muriaux@irim.cnrs.fr).

#### Materials availability

All unique reagents generated in this study are available from the lead contact upon request.

### Method details

Cell lines, cell culture and reagents Vero E6 cells were maintained in Dulbecco’s minimal essential medium (DMEM) supplemented with 10% heat inactivated FBS, 50 U/mL of penicillin, 50 μg/mL of streptomycin, 25 mM of HEPES at 37°C with 5% CO_2_. The human pulmonary alveolar A549-hACE2 cells were engineered using a lentiviral vector from Flash Therapeutics (Toulouse, France) to express the human receptor ACE2 and were sorted by FACS in order to obtain a A549-hACE2 population stably expressing hACE2. Calu-3 cells (epithelial lung adenocarcinoma cells) were maintained in DMEM supplemented with 10% heat inactivated FBS, 50U/mL of penicillin, 50 μg/mL of streptomycin, 25 mM of HEPES at 37°C with 5% CO_2_. U2OS cells were cultivated in DMEM supplemented with 10% heat inactivated FBS, 50 U/mL of penicillin, 50 μg/mL of streptomycin.

#### Screening methodology

For the screening of pharmacological compounds, U2OS cells were cultured in Mc’Coy5A supplemented with 10% fetal bovine serum and 1% penicillin/streptomycin, at 37°C and 5% CO2. Cells were then seeded in a 384-well plate at a density of 3500 cells/well using Multi-Drop combi, in 40 μL of media. For replicate experiments, cells were thawed 10 days before seeding and passed once. The same batch of cryopreserved cells was used for both replicates. Cells were counted with a T4 Cellometer cell counter. After 48 h, the cells were treated with the FDA-approved chemical library from Prestwick Chemicals (1,270 non-patent drugs fully approved by the FDA and other agencies). Cells were pre-incubated for 90 min with drugs at 10 μM concentration and then stimulated for 12 min with IL-1 (10 ng/mL). Cell media was then removed and cells were incubated with cold saponin extraction buffer (80 mM PIPES, 1 mM MgCl2, 1 mM EGTA, 0.1% Saponin, pH 6.8) for 5 min. Finally, cells were fixed with 4% PFA for 15 min, and washed with PBS. All steps were done using the Multi-Channel Arm 384.

NEMO was then labeled by immunofluorescence using the IKKγ antibody (SantaCruz, sc-8330). Cell nuclei were stained with 0.2 μg/mL DAPI. Images of DAPI and Cy3 channels (6 fields/per well) were acquired with an INCell 2000 automated wide-field system at 20× magnification. The experiment was carried out in triplicates. Automated image segmentation was performed using Columbus server, followed by quantitative analysis, resulting in the identification of drug “hit” compounds. Statistical analyses of the results were performed as previously described (Mellouk et al., 2014). The strictly standardized mean difference (SSMD) statistical tests were used for quality control and hit validation analysis. The 4 compounds shown in Table 1 were the strongest hits identified in the screen and were further re-validated through examination of their ability to inhibit the formation of NEMO-containing complexes after IL-1 and TNF stimulation (by immunofluorescence analysis) and through their ability to inhibit the degradation of IκBα after IL-1 stimulation (by immunoblot analysis).

#### Cell lines constructions

##### A549-hACE2-mScarlet-1

A549 were transduced with VSVg pseudotyped particles derived from lentiviral vector from Institut Pasteur (Paris, France) to express the human receptor ACE2 fused with mScarlet-1. Cells were then sorted by flow cytometry (BD FACSAria III Cell Sorter) in order to obtain an A549-hACE2mScarlet-1 population stably expressing hACE2-mScarlet-1t1. MScarlet-1 expression was monitored by flow cytometry in comparison with A549 cells alone. A549-hACE2-mScarlet-1 were maintained in RPMI (Ozyme, France) supplemented with 10% heat-inactivated fetal bovine serum (FBS), 50 U/mL of penicillin, 50 μg/mL of streptomycin, 25 mM of HEPES and 1 mM of sodium pyruvate at 37°C with 5% CO2.

Calu-3 cells (epithelial lung adenocarcinoma cells) were maintained in DMEM supplemented with 10% heat inactivated FBS, 50U/mL of penicillin, 50 μg/mL of streptomycin, 25 mM of HEPES at 37°C with 5% CO_2_.

#### Cell lysis, immunoprecipitation and immunoblotting

Cells were lysed in a buffer containing 50 mM Tris, pH 7.5, 150 mM NaCl, 1% Triton X-100, 1 mM EDTA, and a mixture of protease and phosphatase inhibitors. Protein concentrations in lysates were measured using Bradford assay. For immunoprecipitation of the TNF-R-signaling complex, lysates from FLAG-TNF-treated cells were submitted to an immunoprecipitation using anti-FLAG M2 antibodies coupled to agarose beads. Clarified lysates or immunoprecipitates were mixed with 4 X Laemmli buffer containing DTT and the samples were boiled for 10 min. Proteins were separated by SDS-PAGE using 4–12% acrylamide precast gels and transferred to PVDF membranes. Immunoreactive proteins were visualized by chemiluminescence. For the isolation of soluble and insoluble rafts fractions, adapted procedure from that published by Legler et coll. (Legler et al., 2003) was followed. Briefly, 10 × 10^6^ U2OS cells were lysed on ice for 20 min in 200μL of MNX buffer (1% Triton X-100 in 25 mM MES pH 6.5, 150 mM NaCl supplemented with protease and phosphatase inhibitors). The cell lysate was homogenized (10 strokes) with a loose-fitting glass Dounce homogenizer and spun at 800g for 5 min at 4°C to remove the nuclear pellet. The postnuclear supernatant was centrifuged at 100,000 g for 1 h at 4°C. The lipid raft fraction in the pellet was resuspended in 200μL of 0.5% Brij 78. Non-soluble material was removed by an additional centrifugation for 20 min at 16,000g. The 100,000 g supernatant is referred to as the Triton X-100 soluble fraction containing the phospholipid membrane and cytosolic fraction.

#### Fluorescence-activated cell sorting (FACS)

For quantification of cell surface expression of TNF-R1 and IL1-R1, U2OS cells either untreated or treated for 90 min with Auranofin at 2.5 μM or 5 μM, were detached with non-enzymatic dissociate buffer, resuspended in PBS and fixed with 1% PFA. For TNF-R1 detection, an anti-TNFR1-coupled to PE was used in parallel with an isotype control antibody. For IL1-R1 expression, a goat anti-IL1-R1 was used followed by a staining with an anti-goat-Alexa Fluor 488 antibody. Cells stained with anti-goat-Alexa Fluor 488 antibody alone was used as control. FACS analyses were performed on LSRFortessa Cell Analyzer and the results were analyzed with FlowJo 10.0 software.

#### SARS-CoV-2 virus stock and titration

The strain BetaCoV/France/IDF0372/2020 was propagated in Vero E6 cells with DMEM containing 2.5% FBS, 25 mM HEPES at 37°C with 5% CO_2_ and was harvested 72h post inoculation. Virus stocks were stored at −80°C. Virus titration from infected cell culture supernatant were monitored using plaque assays on a monolayer of Vero E6 cells, using 200 μL of virus solution. Samples were serially diluted and the plaque-forming units (PFU) values were determined using crystal violet coloration on cells and subsequent scoring the amounts of wells displaying cytopathic effects. Calculations allow the determination of the titer as the number of PFU/mL.

#### SARS-CoV-2 VLP production

SARS-CoV-2 virus-like particles were made as described (Gourdelier et al., 2022; Swann et al., 2020). Briefly, HEK293T human embryonic kidney cells were transfected with plasmids expressing SARS-CoV-2 M, M(GFP), N, E and S proteins as described (Swann et al., 2020), using phosphate calium method. Transfected cells were washed 6h later with PBS (PBS 1x) and VLP harvested 48h post-transfection. VLPs were obtained from culture medium of transfected cells by clarification at 5,000 rpm for 5 min at 4°C. Then the VLP were collected from clarified supernatants after ultracentrifugation, through a 25% sucrose cushion in TNE buffer (10 mM Tris-HCl pH 7.4, 100 mM NaCl, 1 mM EDTA), at 100,000g for 3h at 4°C in a Beckman SW41Ti rotor. VLPs were resuspended in TNE1x buffer and stored at 4°C.

### Assessment of antiviral activity

Antiviral activity was assessed by lysing the cells at 48h post-infection and proceeds for RT-qPCR to measure viral infectivity. Briefly, 30,000 cells per well were cultured in 96-well plates for 24h. Compounds were diluted from stock to different concentrations for testing antiviral activity on A549-hACE2, Vero E6 and Calu-3 cells. Concentrations range were 5 μM–0.04 μM for Auranofin; 5 μM–0.025 μM for Remdesivir; 10 μM–0.05 μM for Camostat Mesylate; 10 μM–0.05 μM for Parthenolide; MβCD 40 μM–0.18 μM; ML120B 25 μM–0.18 μM. Cells were incubated with 100 μL of the compounds diluted in RPMI, 0.1% DMSO at the indicated concentrations, and the plates were incubated for 2h. Subsequently, cells were either mock infected (for analysis of cytotoxicity of the compound) or infected with 400PFU of virus per well (MOI of 0.01) in a total volume of 110 μL of medium with compound. Cell viability was assessed 2 dpi by a cell proliferation assay (MTS). Absorption was read at 490nm (MTS). The 50% effective concentration (EC_50_, the concentration required to inhibit virus-induced cell death by 50%) and the 50% cytotoxic concentration (CC_50_, the concentration that reduces the viability of uninfected cells to 50% of that of untreated control cells) were determined using 4-parameters nonlinear regression with GraphPad Prism v8.0, based on the following calculations:

$$\text{Cell}\;\text{cytotoxicity},\;\%\text{TOX}=\left(1-\frac{drug}{cell\;DMSO}\right)\times 100$$

$$\text{Percentage}\;\text{of}\;\text{viral}\;\text{replication}\;\text{inhibition},\;\%\text{Inhibit}=\left(\frac{drug-\text{infected}\;\text{cells}}{cell\;DMSO-\text{infected}\;\text{cells}}\right)\times 100$$

Data are mean ± SD of three biological replicates, each of which consisted of duplicate samples. Data were normalized as the relative efficiency or cell viability of inhibitor-treated cells compared with those of untreated cells (set to 100%).

#### Quantitative reverse transcription polymerase chain reaction (RT-qPCR)

Quantitative RT-PCR was carried out in triplicate, on 1μL of purified RNA. 2 sets of primers were used, the first targets the SARS-CoV-2 E gene (E_Sarbeco-F ACAGGTACGTTAATAGTTAATAGCGT; E_Sarbeco-R ATATTGCAGCAGTACGCA CACA). The second primer targets the GAPDH gene (GAPDH For: GCTCACTGGCATGGCCTTCCGTG; GAPDH Rev: TGGAGGAGTGGGTGTCGCTGTTG) and was used to standardize the data obtained. Viral RNAs were isolated from infected cells 48h post infection using the Luna Cell Ready Lysis Module. Viral RNAs were quantified by RT-qPCR in triplicate as described in (Corman et al., 2020).

#### Immunofluorescence and confocal microscopy infected cell imaging

A549-hACE2 cells seeded on glass coverslips were infected with SARS-CoV-2 at a MOI of 0.01. At 48h pi, cells were washed with PBS and fixed in 4% paraformaldehyde in PBS for 30 min at room temperature, followed by permeabilization with 0.2% Triton X-100 in PBS for 5 min, washed and blocking in 2% BSA in PBS for 15 min. Incubation with primary antibodies anti-SARS-CoV-2 rabbit membrane (M) protein (1:100) was performed for 2h at RT. After washing with PBS, cells were incubated with secondary antibodies AF568-labeled goat-*anti*-rabbit (1:2000) for 2h at RT and subsequent staining with Phalloidin-AX488 (1:200) for 1h at RT for F-actin labeling. Coverslips were sealed with Prolong Diamond Antifade reagent. Confocal fluorescence images were generated using a LSM780 confocal laser-scanning microscope equipped with a 63X, 1.4 NA oil objective.

For non-infectious immunofluorescence, cells were cultured on glass coverslips, fixed with either 4% PFA or 100% methanol. After fixation in methanol, cells were progressively rehydrated by successive washes with 95%, 80%, and 50% ethanol in water and two final washes with PBS. PFA-fixed cells were permeabilized with 0.2% Triton X-100. After blocking by incubation with 1% BSA in PBS, the slides were incubated with the primary antibodies, washed with PBS, incubated with species-specific fluorochrome-tagged secondary antibodies, washed and incubated with DAPI to stain the nuclei. The coverslips were mounted in Mowiol or Prolong Diamond Antifade reagent. Image analyses were performed with either an ApoTome imaging system equipped with a 63×/1.4 NA oil differential interference contrast (DIC) objective lens or a confocal microscope (LSM700) equipped with a 63× Plan Apochromat/1.4 NA oil objective lens. Images were acquired and analyzed with AxioVision or Zen software. Quantification analysis of EGF internalization was performed using CellProfiler software.

#### Statistical analyses of immunofluorescent experiments

Statistical tests were performed using Origin 19 software. Statistically significant analyses were evaluated using one-way ANOVA and t tests. Quantification analysis were performed using ImageJ/Fiji software.

#### Sucrose density gradient centrifugation

Detergent-resistant membranes (DRM) were produced from 15 × 10^6^ cells using Triton X-100 lysis and isolated by sucrose density gradient ultracentrifugation according to Jouannet et al. (2016). Briefly, 15 × 10^6^ cells were lysed in 250 μL of lysis buffer (20 mM Tris pH 7.5, 1% Triton X-100, 150 mM NaCl), containing protease inhibitor cocktail for 30 min on ice. 200 μL lysate was then mixed with 800 μL of 60% sucrose (prepared in lysis buffer with detergent, supplemented with protease inhibitor), and placed on the bottom of 5mL ultracentrifuge tube (344,057, Beckman). The sample was overlaid with 2.5mL of 30% sucrose and 1mL of 2,5% sucrose (prepared in lysis buffer without detergent, supplemented with protease inhibitor) and centrifuged at 200,000 g for 16 h at 4°C in swing buckets of MLS-50 rotor using an Optima MAX-XP ultracentrifuge. After ultracentrifugation, 9 fractions of 0.5mL were collected from top (low sucrose concentration) to bottom of the tube (high sucrose concentration). Fractions were analyzed by immunoblot analysis.

#### Cholesterol measurements and ^3^H cholesterol efflux assay

Cholesterol was measured using the Amplex Red cholesterol assay kit. This biochemical assay uses an enzyme-coupled reporting system for the detection of cholesterol concentrations in samples. Cell fractionations were performed to measure cholesterol content in cell membranes. Membrane preparations from Vero-E6 cells were done as follows: after cell detachment in PBS, 8 × 10^6^ cell were resuspended in hypotonic buffer containing 10 mM HEPES (pH 7.9) 1.5 mM MgCl2, 10 mM KCl, 0.5 mM DTT and protease inhibitors. After dounce homogenization (20 strokes), nuclei were removed by a 800g centrifugation for 5 min. Supernatants were collected and centrifuged for 1h at 100,000g using a TLA 100.3 fixed-angle rotor (Beckman) in an Optima MAX-XP ultracentrifuge (Beckman). Membrane pellets were dissolved in Amplex Red assay buffer and normalized to protein concentrations (Bradford assay). Amplex Red assay was done according to manufacturer’s protocol. Fluorescence was measured in a fluorescence microplate reader using excitation at 560nm and emission detection at 590nm. Median fluorescence intensity in the control sample (assay buffer only) was subtracted from the measured intensities.

Cholesterol efflux assay using cultured cells was performed as reported previously (Catalano et al., 2008). 50,000 cells were seeded in triplicates in 24 wells plates. 24h after, cells were incubated with DMEM containing 1 μCi/mL ^3^H-cholesterol for 24h. Cells were then washed twice with DMEM, followed by equilibration with DMEM containing 0.2% BSA and either left untreated or treated with 2.5 μM Auranofin for 24h. Alternatively, cells were treated for 4h with 5 μM Auranofin or 2 mM MβCD. Cholesterol efflux was assessed in DMEM medium containing 2,5% human serum (1–40 dilution, 4 h) coming from a normo-lipidemic subject. The radioactivity in the extra-cellular medium and cells were determined by scintillation counting. Cholesterol efflux capacity (CEC) was calculated using the following formula: ^3^H-cholesterol in medium/(^3^H-cholesterol in medium + ^3^H-cholesterol in cells) × 100.

#### Effect of Auranofin on lipid organization in cell membranes

Cells were seeded in triplicate on a 48-well plate. Two days later, cells were either untreated or treated with increasing concentrations of MβCD or Auranofin in medium without phenol red. Plates were equilibrated at room temperature and NR12S was added to the well at 40 nM final concentration for 7 min in the dark. After 3 washes with DMEM, fluorescence was measured using a FlexStation 3 microplate reader. Excitation was set at 520nm and fluorescence emission was recorded at 580 and 630 nm. Background fluorescence (without prior incubation with NR12S) was subtracted from the measured values.

#### Spot variation fluorescence correlation spectroscopy (svSCF) measurements on cells

Auranofin (stock at 5 mM) was diluted 1/100 in Tris-HCl Buffer. FCS was measured on untreated and 5 μM Auranofin treated A549-hACE2-mScarlet-1 cells at 37°C. After 1h, svFCS measurement was processed on live cells under a LSM780 confocal laser-scanning microscope (Zeiss). HeNe 561-nm excitation is used for mScarlet-1 fluorophore. Before running the experiment, a 1 μM Rhodamine solution was used to adjust the pinhole, and FCS measurements were done at different pinhole openings. The τ_D_ (diffusion time) measures are used to calculate the waist ω^2^ at each pinhole opening (see the equation of General FCS diffusion law: τ_D_ = ω^2^/4D+ τ(D_0_)) and a correspondence between pinhole opening and waist is drawn. Laser power is adapted to reach CPP (count per particle) values around 4kHz for each measurement. Data analysis is processed using PyCorrFit and GraphPad Prism 9 software. The behavior of fluorescent molecules within several membranes’ organizations using general FCS Diffusion law has previously been described (Lenne et al., 2006).

#### Cell-cell fusion assay

HEK 293T cells were transduced to express either the GFP 1–10 or the GFP11 half of the GFP. GFP11-expressing or GFP1-10-expressing HEK 293T cells were transduced to stably express the SARS-CoV-2 Spike protein or the ACE2 receptor, respectively. HEK 293T-S-GFP11 and HEK 293T-ACE2-GFP1-10 cells were mixed at a 1:1 ratio and plated at 6 × 10^4^ total cells per well in a μClear 96-well plate in the presence of increasing concentrations of Auranofin. After 48h, images were acquired with an Opera Phenix high-content confocal microscope and the GFP area as well as the number of nuclei was quantified using the Harmony software.

#### Endocytosis assays

Cells were seeded in 24-well plates containing glass coverslips for 2–3 days in order to reach 80% confluency. Cells were pretreated with Auranofin for 90 min at the indicated concentration. After medium wash containing 0.5% FCS, cells were incubated for 40 min at 4°C in medium containing 0.5% FCS, 20 mM HEPES pH7.0, Auranofin at the desired concentration and either biotinylated-EGF/streptavidin-Alexa555 (650 ng/mL), choleratoxine-FITC (2.5 μg/mL) or Transferrin-A488 (1 μg/mL). After a 0.5% FCS-containing medium wash, cells were incubated at 37°C for the indicated time in FCS-containing medium supplemented with Auranofin. Cells were then washed twice with PBS and fixed with 4% PFA. After a wash in PBS, cells were permeabilized with 0.2% Triton X-100 (5 min) and the nuclei were stained with DAPI. After two washes in PBS, coverslips were mounted with Mowiol (Biovalley). For the quantification of EGF internalization, cells were further stained with CellMask-Cy5 dye (200 ng/mL, 30 min incubation) in order to detect cell borders. Quantification was done using CellProfiler software using a minimum of n = 300 cells per condition.

#### Fluorescent VLP internalization assay

Virus particle internalization assays were performed using M(GFP) labeled MNES SARS-CoV-2 VLP. After 24h culture on glass coverslip, A549-hACE2_mScarlet-1_ cells were harvested and incubated at 37°C for 1h with or without 5 μM Auranofin. VLP(GFP)-MNES were then diluted at 1:20 ratio in red phenol-free DMEM and incubated with processed A549-hACE2_mScarlet-1_ cells. Unbound VLPs were removed by 3 washes in PBS. Cells were fixed with 4% PFA and mounted with Prolong gold antifade agent with DAPI for confocal imaging. Confocal fluorescence images were generated using a LSM780 confocal laser-scanning microscope equipped with a 63X, 1.4 NA oil lens. The cells were imaged with 0.3 μm section Z stacks. Z-projected (max intensities) images were analyzed using ImageJ/Fiji software.

## Data Availability

All data, code, and materials used in the manuscript are available to any researcher for purposes of reproducing or extending the analyses. All data are available in the main text or in the supplemental information. The data obtained from the drug screen are available at: https://doi.org/10.7910/DVN/DS3R8N. Cell lines, cell culture and reagents: Vero E6 cells were maintained in Dulbecco’s minimal essential medium (DMEM) supplemented with 10% heat inactivated FBS, 50 U/mL of penicillin, 50 μg/mL of streptomycin, 25 mM of HEPES at 37°C with 5% CO_2_. The human pulmonary alveolar A549-hACE2 cells were engineered using a lentiviral vector from Flash Therapeutics (Toulouse, France) to express the human receptor ACE2 and were sorted by FACS in order to obtain a A549-hACE2 population stably expressing hACE2. Calu-3 cells (epithelial lung adenocarcinoma cells) were maintained in DMEM supplemented with 10% heat inactivated FBS, 50U/mL of penicillin, 50 μg/mL of streptomycin, 25 mM of HEPES at 37°C with 5% CO_2_. U2OS cells were cultivated in DMEM supplemented with 10% heat inactivated FBS, 50 U/mL of penicillin, 50 μg/mL of streptomycin.
