# Supplementary material for: The FDA-approved drug Auranofin has a dual inhibitory effect on SARS-CoV-2 entry and NF-κB signaling
Source: iScience. 2022 Sep 3;25(10):105066. doi: 10.1016/j.isci.2022.105066 (PMC9439859; doi:10.1016/j.isci.2022.105066)
Supplement: Document S1. Figures S1–S10 [file mmc1.pdf]

iScience, Volume *25*

Supplemental information

The FDA-approved drug Auranofin has a dual inhibitory effect on SARS-CoV-2 entry and NF-κB signaling

Emmanuel Laplantine, Christine Chable-Bessia, Anne Oudin, Jitendryia Swain, Adèle Soria, Peggy Merida, Manon Gourdelier, Sarra Mestiri, Indira Besseghe, Erwan Bremaud, Aymeric Neyret, Sebastien Lyonnais, Cyril Favard, Philippe Benaroch, Mathieu Hubert, Olivier Schwartz, Maryse Guerin, Anne Danckaert, Elaine Del Nery, Delphine Muriaux, and Robert Weil

Fig. S1. Auranofin does not modify TNF-R1 and IL1-R1 expression

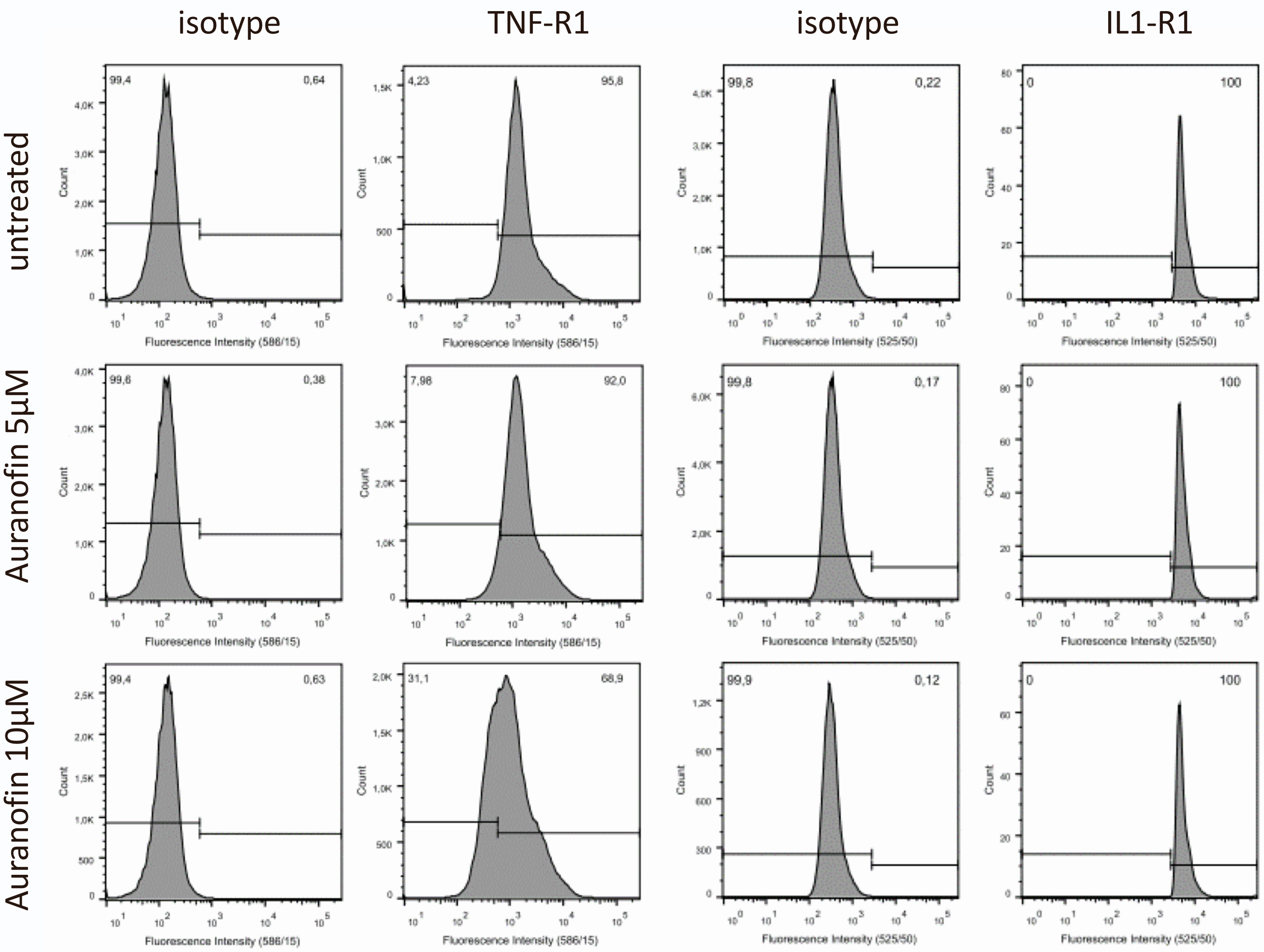

Fig. S2. Effects of the IKK inhibitor ML120B on NF-κB activation pathway

A

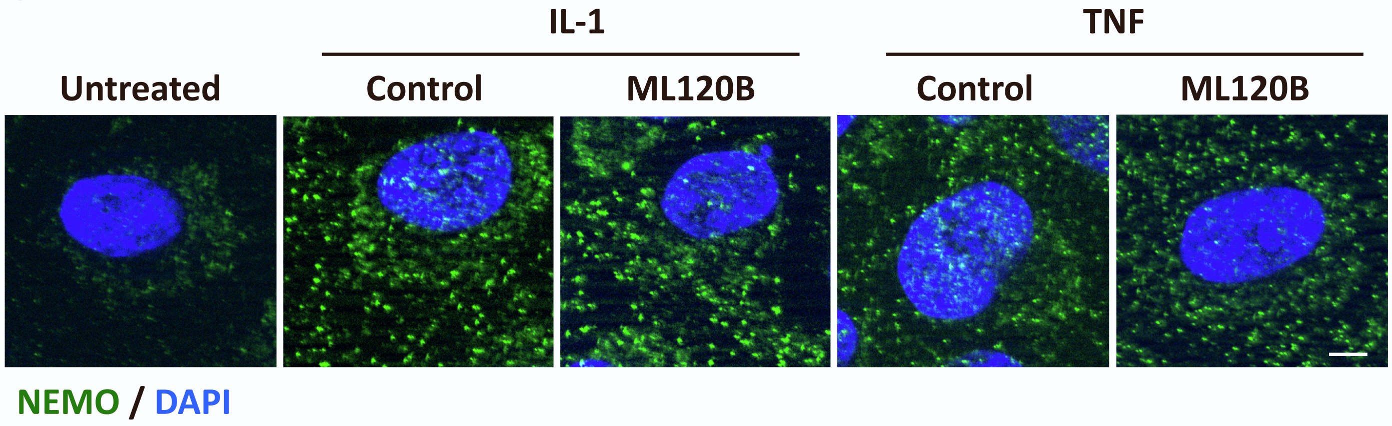

B

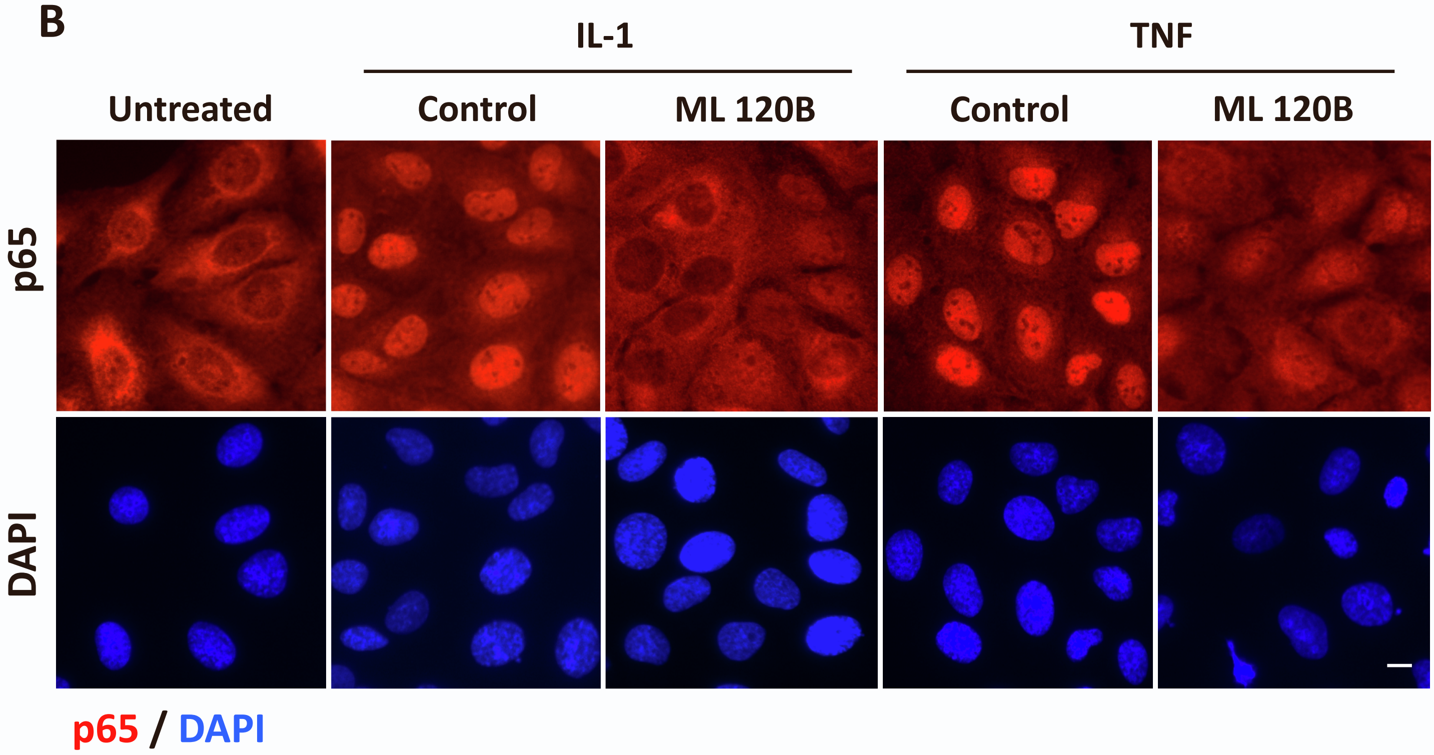

Fig. S3. Auranofin inhibits the recruitment of IRAK1 and NEMO to supramolecular complexes in response to IL-1

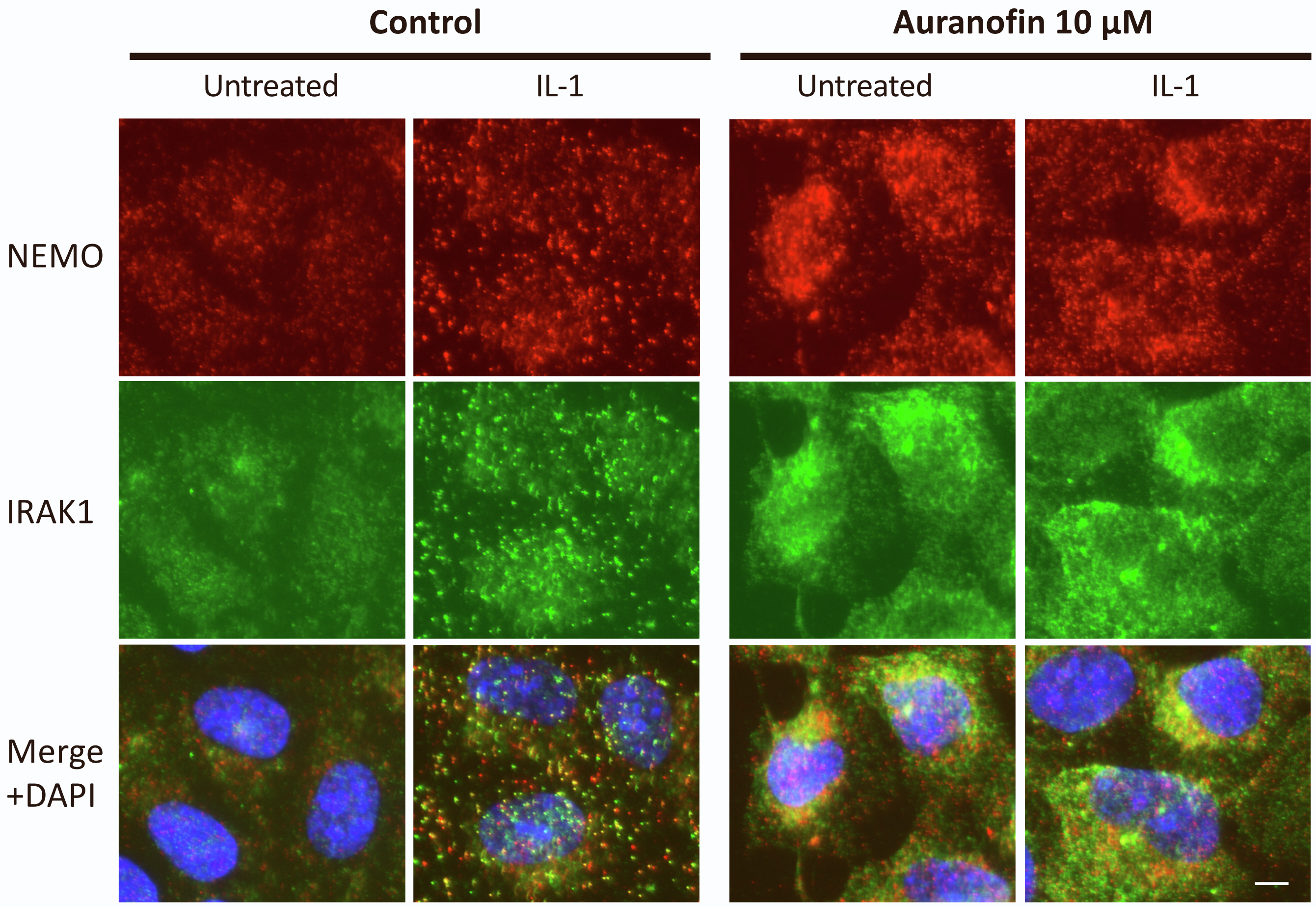

Fig. S4. Auranofin inhibits the innate antiviral response after SARS-CoV-2 infection

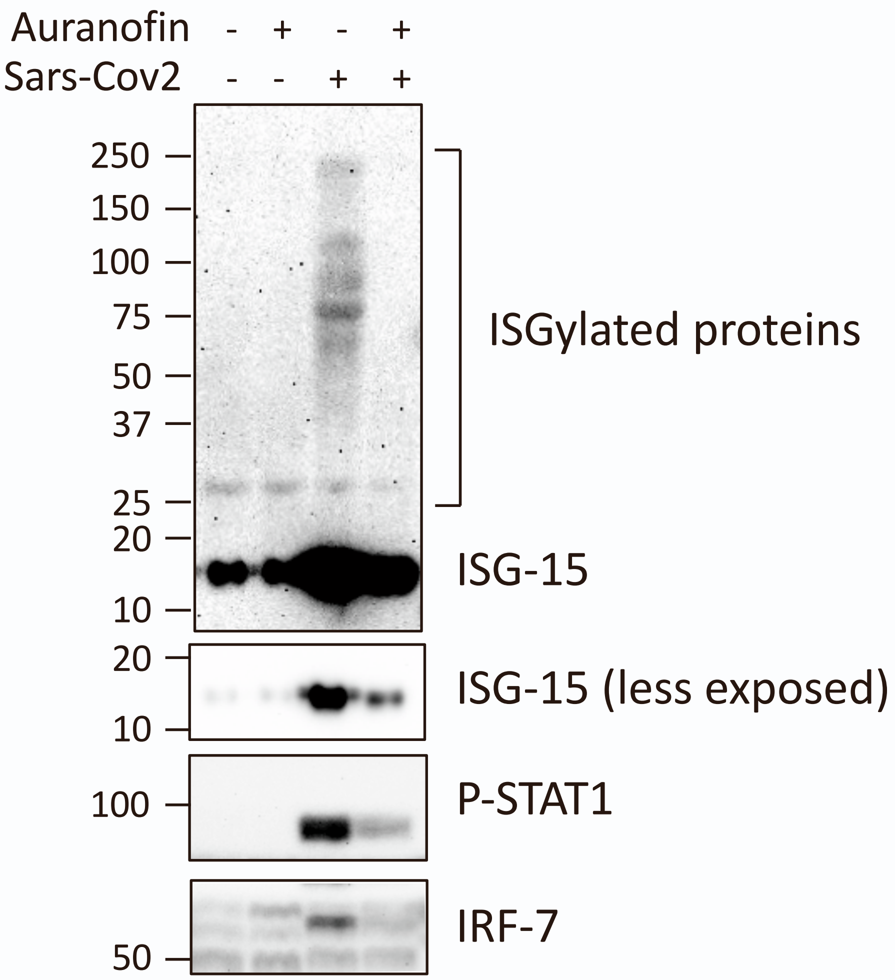

Fig. S5. Expression of hACE2 and TMPRSS2 in different cell lines used in this study

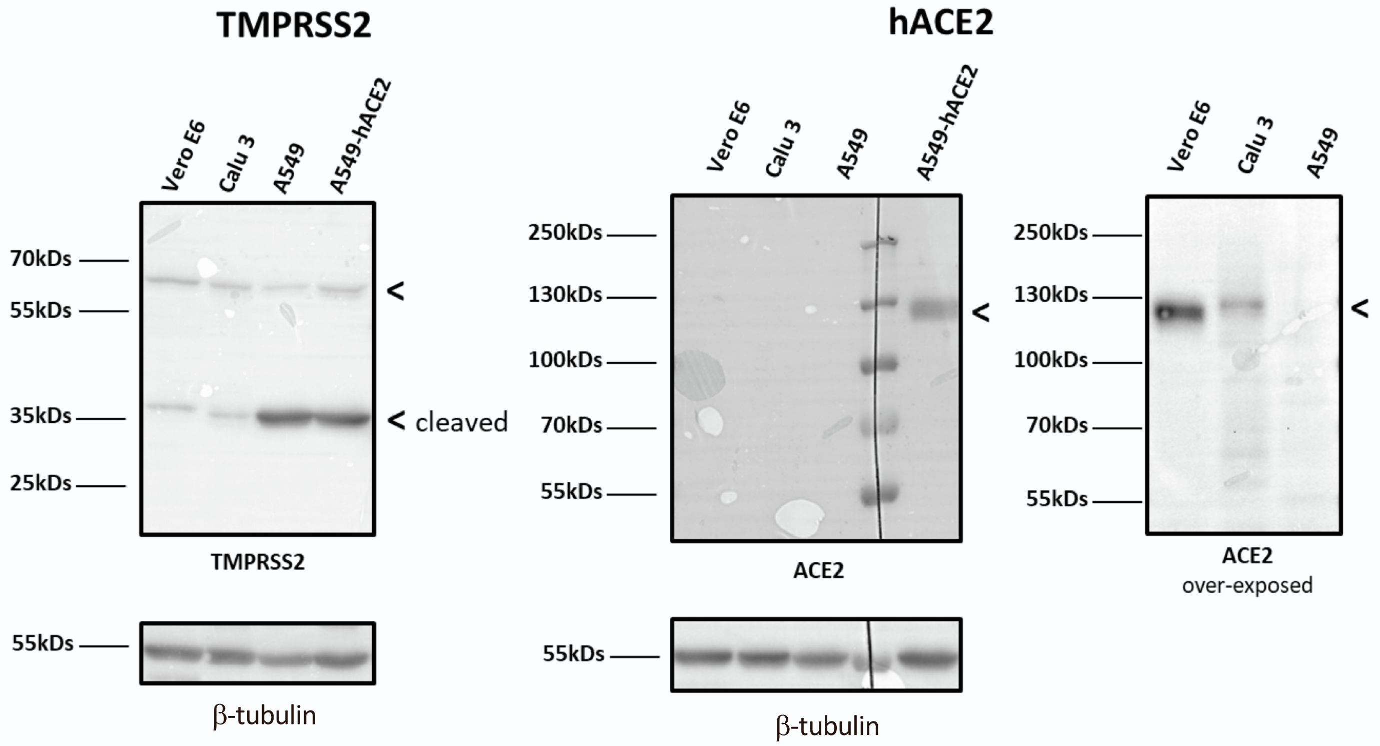

Fig. S6. Dose-dependent response of Partenolide, ML120B and MβCD on SARS-CoV-2 infected Vero E6 cell lines

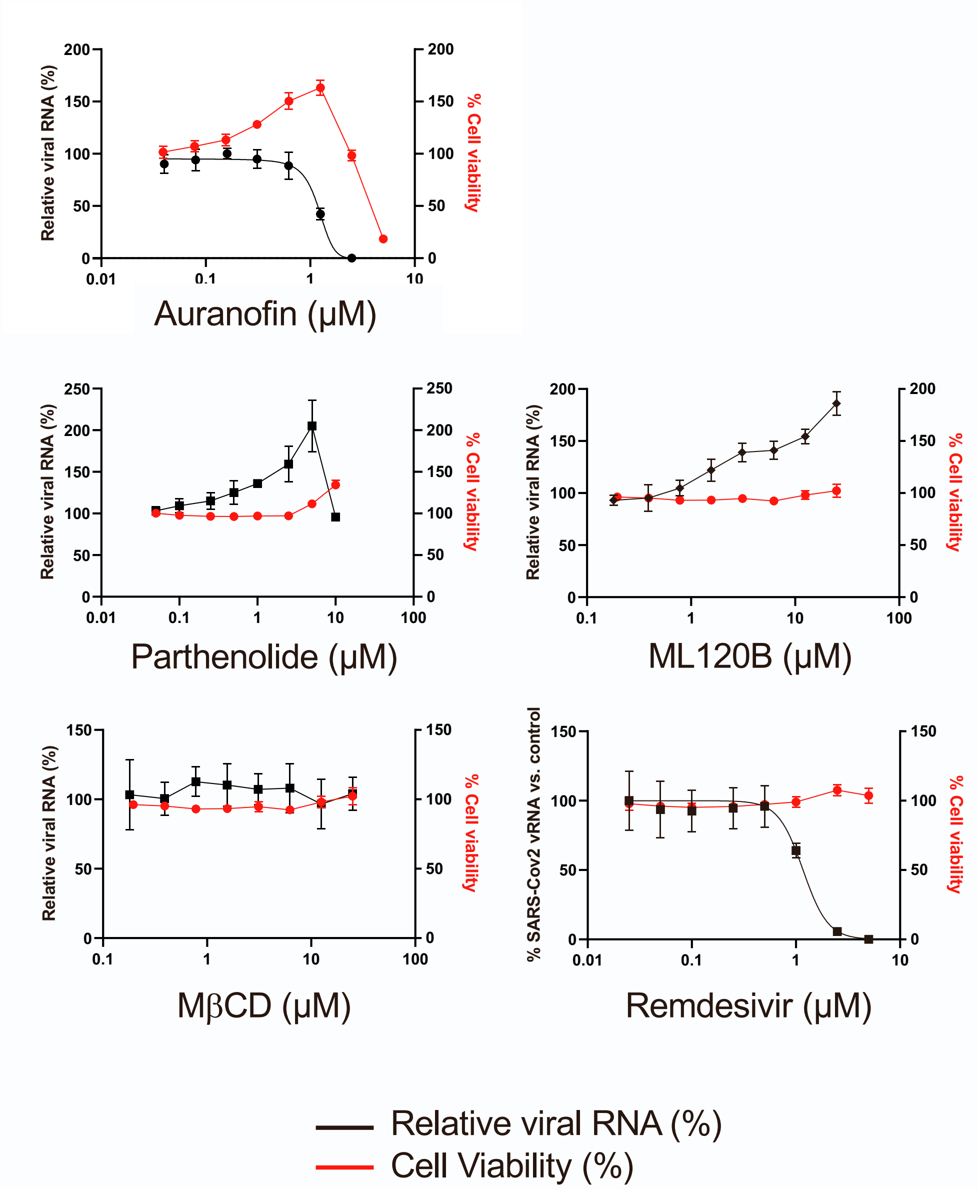

Fig. S7. Auranofin effect on cholesterol

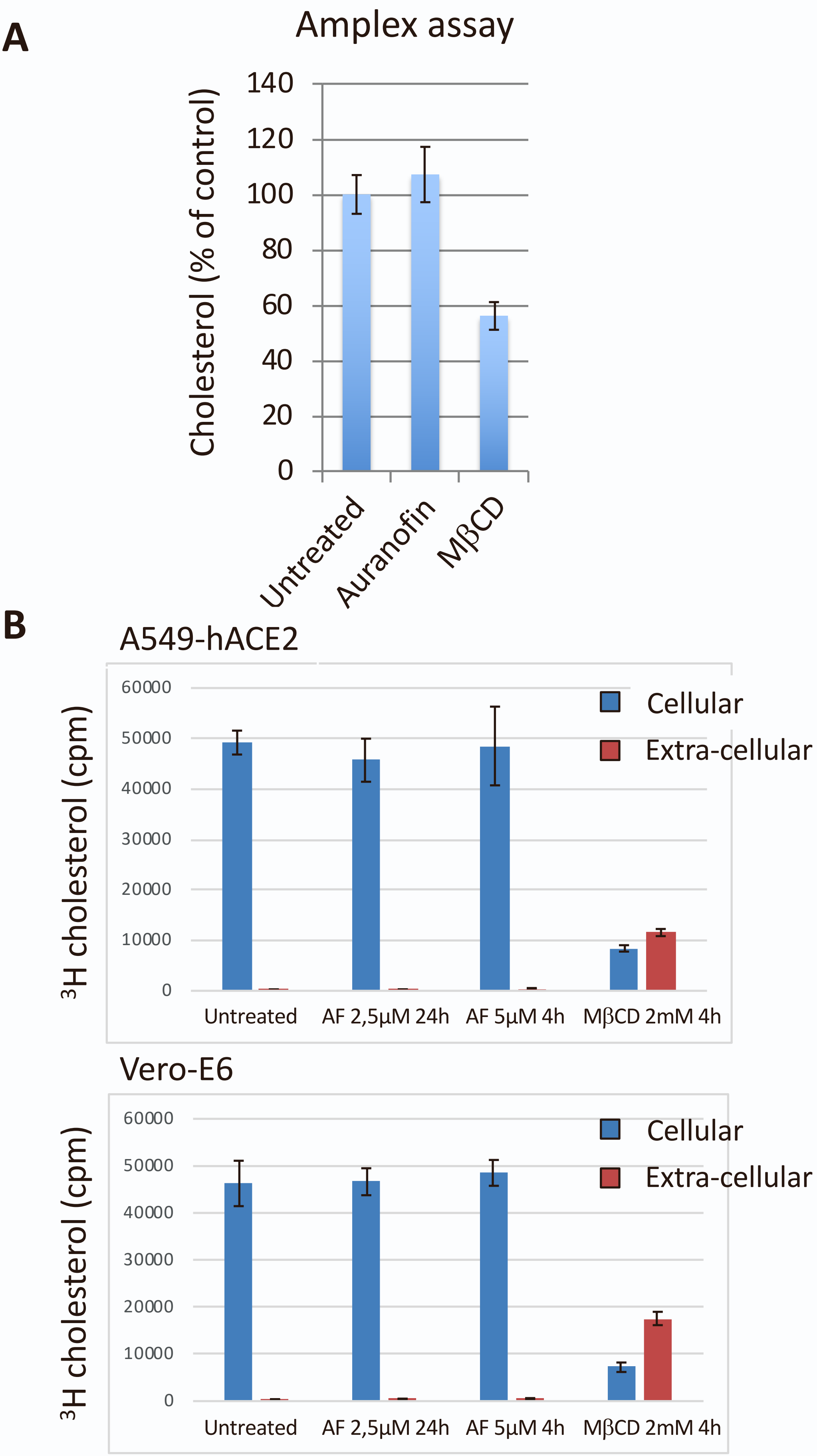

C

EFFLUX (with serum)	A549-hACE2	VERO-E6
**Ctrl**	6 %	10%
**Auranofin 2.5µM 24h**	5 %	11%
**Auranofin 5µM 4h**	5 %	11%

Fig. S8. Auranofin disturbs the lipid order of the plasma membrane

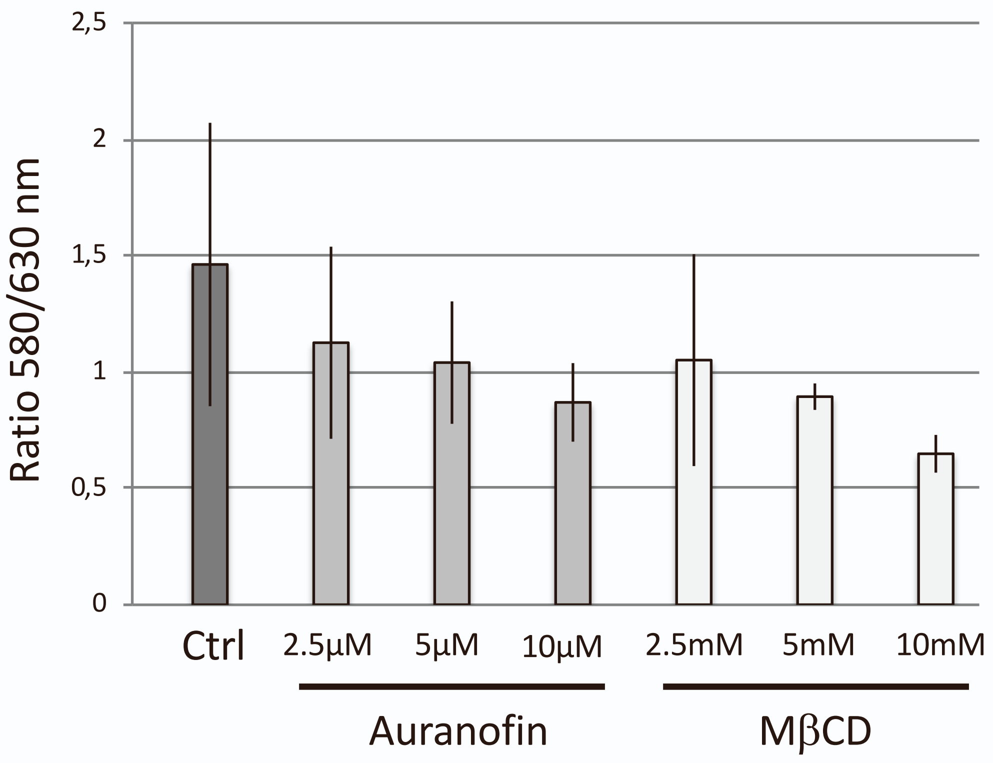

Fig. S9. Effect of Auranofin on SARS-CoV-2 Spike-mediated cell-cell fusion

A

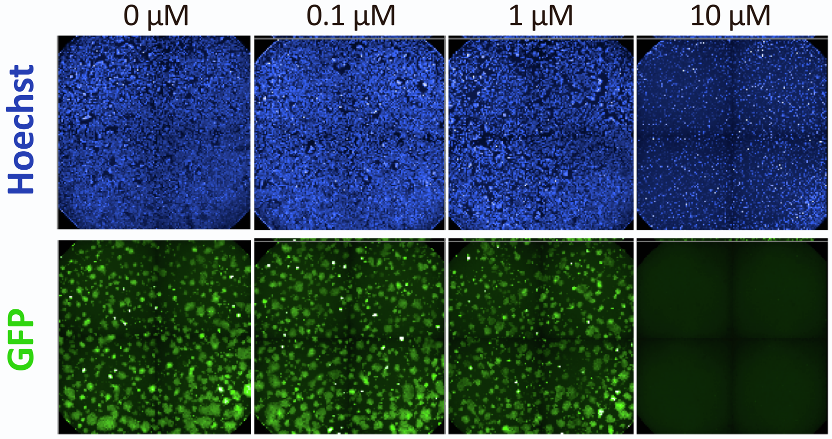

B

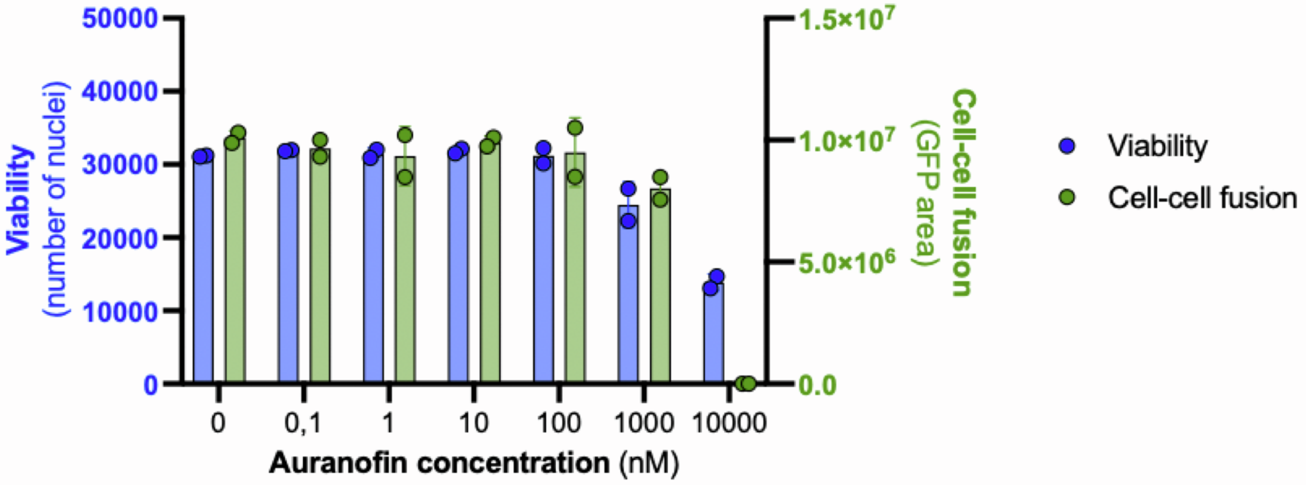

Fig. S10. Auranofin does not inhibit Transferrin endocytosis

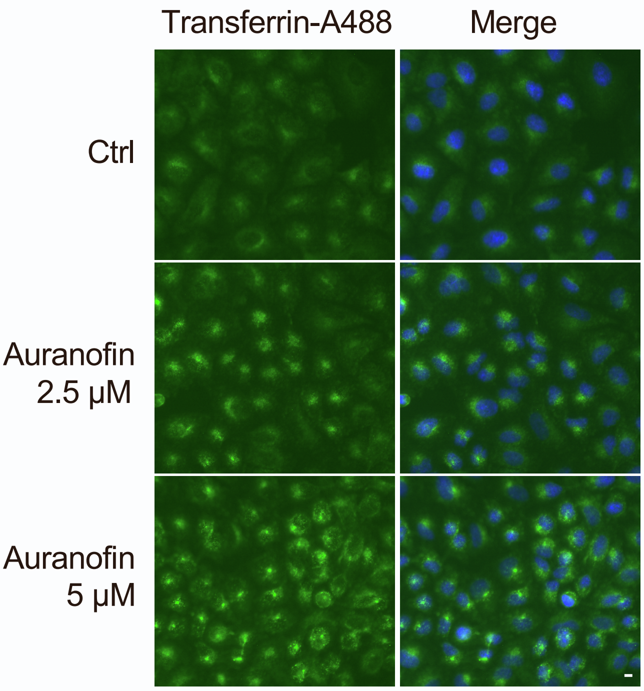

Supplemental Figures legends

Figure S1: **Auranofin does not modify TNF-R1 and IL1-R1 expression.**

Flow cytometry determination of TNF-R1 and IL1-R1 expression levels. Fluorescence intensity of TNF-R1 and IL1-R1 membrane expression using a relevant isotype antibody as negative control in either untreated or Auranofin-treated U2OS cells (concentrations are indicated). x-axis is Fluorescence intensity and y-axis is Count numbers.

Related to Figure 2.

Figure S2: **Effects of the IKK inhibitor ML120B on NF-κB activation pathway.**

(A) Inhibition of IKK activity prevents nuclear translocation of p65. U2OS cells were pretreated with ML120B and stimulated with IL-1 or TNF-α as indicated. p65 was stained by immunofluorescence and visualized by microscopy. Nuclei were stained with DAPI. Scale bar, 5 μm. **(B)** Inhibition of IKK kinase activity does not prevent the recruitment of NEMO in supramolecular structures. U2OS cells were pretreated with ML120B and stimulated with IL-1 or TNF-α as in (A) and the recruitment of NEMO (green) in the supramolecular complexes was visualized by immunofluorescence. Nuclei were stained with DAPI. Scale bar, 5 μm.

Related to Figure 2.

Figure S3: **Auranofin inhibits the recruitment of IRAK1 and NEMO to supramolecular complexes in response to IL-1.**

Immunofluorescence analysis of the effect of Auranofin on NEMO- and IRAK1-containing supramolecular complexes in U2OS stimulated with IL-1. NEMO (in red) and IRAK1 (in green) were stained by immunofluorescence. Nuclei were stained with DAPI. Scale bar, 5 μm.

Related to Figure 2.

Figure S4: **Auranofin inhibits the innate antiviral response after SARS-CoV-2 infection.**

CALU-3 cells were pre-incubated with 2,5 μM Auranofin for 1h30 before infection with SARS-CoV-2 at MOI 0.05 for 48 hours. Whole cell lysates were immunoblotted with anti-ISG15, anti-STAT1 and anti-IRF3 antibodies.

Related to Figure 2.

Figure S5: **Expression of hACE2 and TMPRSS2 in different cell lines used in this study.**

(A) Immunoblots using anti-TMPRSS2 on cell lysates showing higher TMPRSS2 expression in human pulmonary A549-hACE2 cells compared to simian Vero E6 and human Calu-3 cell lines. **(B)** Immunoblots using anti-hACE2 showing strong hACE2 expression in A549-hACE2

cell line. An over-exposition of the same membrane shows the presence of hACE2 in Vero E6 and Calu-3 cells as well. Tubulin was used as loading control.

Related to Figure 3.

Figure S6: **Dose-dependent response of Partenolide, ML120B and MβCD on SARS-CoV-2 infected Vero E6 cell lines.**

Red line: cell toxicity using MTS. Black Line: cell lysates 48h post-infection (MOI of 0.01) were used for qRT-PCR of the SARS-CoV-2 E gene to evaluate the antiviral activity (n=3 independent experiments in duplicate). On this figure, Auranofin and Remdesivir graphs, which are a compilation of several experiments, have been duplicated from Figure 3.

Related to Figure 3 and Table 1.

Figure S7: **Auranofin effect on cholesterol.**

(A) Membrane cholesterol levels were quantified using the Amplex Red cholesterol Assay kit. Plasma membrane preparations from Vero E6 cells were used to measure the level of cholesterol in cells either untreated, treated 25 min with 10 mM MβCD or treated 16h with 2.5μM Auranofin as indicated. **(B)** Vero E6 and A459 hACE2 cells were incubated for 24h with H^3-labeled Cholesterol (1μCi/ml), washed and incubated without or with Auranofin (2.5 μM or 5 μM) or MβCD (2 mM) for 4 or 24 hours in serum-free medium as indicated. Radioactivity in cells and in the media was measured using a scintillation counter. **(C)** For cholesterol efflux measurements, cells were incubated during the last 4 hours with 2.5% human serum or left untreated. Radioactivity in cells and in the media was measured as mentioned above using a scintillation counter. Data are mean ± SD for each condition done in triplicate.

Related to Figure 4.

Figure S8: **Auranofin disturbs the lipid order of the plasma membrane.**

U2OS cells were treated with MβCD or Auranofin at the indicated concentration for 90 min. Cells were then incubated with 40nM NR12S for 7 min in the dark, washed and a spectrophotometer was used to excite at 520nm and measure the fluorescence emission of NR12S at 580nm and 630nm. Ratio of emission 580/630 was then calculated.

Related to Figure 4.

Figure S9: **Effect of Auranofin on SARS-CoV-2 Spike-mediated cell-cell fusion.**

(A) HEK 293T cells stably expressing the SARS-CoV-2 Spike protein or ACE2 receptor and harboring the GFP-split system were co-cultured at a 1:1 ratio in the presence of increasing concentrations of Auranofin. 48h later, GFP^+ syncytia and the number of nuclei were quantified

by confocal microscopy. (**B)** Quantification of the Hoechst and GFP signals as markers of the cell viability and cell-cell fusion, respectively. Results were expressed as mean SD

Related to STAR Methods.

Figure S10: **Auranofin does not inhibit Transferrin endocytosis.**

A549-hACE2 cells treated or not with Auranofin at the indicated concentrations for 2h were stimulated with Alexa Fluor 488-coupled Transferrin for 15 min at 37°C. Cells were then fixed in 4% paraformaldehyde, stained with DAPI and analyzed by microscopy. Scale bar, 5 μm.

Related to Figure 6.
